# Heterogeneity in HIV and cellular transcription profiles in cell line models of latent and productive infection: implications for HIV latency

**DOI:** 10.1186/s12977-019-0494-x

**Published:** 2019-11-11

**Authors:** Sushama Telwatte, Sara Morón-López, Dvir Aran, Peggy Kim, Christine Hsieh, Sunil Joshi, Mauricio Montano, Warner C. Greene, Atul J. Butte, Joseph K. Wong, Steven A. Yukl

**Affiliations:** 1 San Francisco VA Medical Center, San Francisco, CA USA; 2 University of California San Francisco, San Francisco, CA USA; 3 Bakar Computational Health Sciences Institute, University of California, San Francisco, San Francisco, CA USA; 4 Gladstone Institute of Virology and Immunology, San Francisco, CA USA

**Keywords:** HIV-1, Transcription, Latency, Latent infection

## Abstract

**Background:**

HIV-infected cell lines are widely used to study latent HIV infection, which is considered the main barrier to HIV cure. We hypothesized that these cell lines differ from each other and from cells from HIV-infected individuals in the mechanisms underlying latency.

**Results:**

To quantify the degree to which HIV expression is inhibited by blocks at different stages of HIV transcription, we employed a recently-described panel of RT-ddPCR assays to measure levels of 7 HIV transcripts (“read-through,” initiated, 5′ elongated, mid-transcribed/unspliced [Pol], distal-transcribed [Nef], polyadenylated, and multiply-sliced [Tat-Rev]) in bulk populations of latently-infected (U1, ACH-2, J-Lat) and productively-infected (8E5, activated J-Lat) cell lines. To assess single-cell variation and investigate cellular genes associated with HIV transcriptional blocks, we developed a novel multiplex qPCR panel and quantified single cell levels of 7 HIV targets and 89 cellular transcripts in latently- and productively-infected cell lines. The bulk cell HIV transcription profile differed dramatically between cell lines and cells from ART-suppressed individuals. Compared to cells from ART-suppressed individuals, latent cell lines showed lower levels of HIV transcriptional initiation and higher levels of polyadenylation and splicing. ACH-2 and J-Lat cells showed different forms of transcriptional interference, while U1 cells showed a block to elongation. Single-cell studies revealed marked variation between/within cell lines in expression of HIV transcripts, T cell phenotypic markers, antiviral factors, and genes implicated in latency. Expression of multiply-spliced HIV Tat-Rev was associated with expression of cellular genes involved in activation, tissue retention, T cell transcription, and apoptosis/survival.

**Conclusions:**

HIV-infected cell lines differ from each other and from cells from ART-treated individuals in the mechanisms governing latent HIV infection. These differences in viral and cellular gene expression must be considered when gauging the suitability of a given cell line for future research on HIV. At the same time, some features were shared across cell lines, such as low expression of antiviral defense genes and a relationship between productive infection and genes involved in survival. These features may contribute to HIV latency or persistence in vivo, and deserve further study using novel single cell assays such as those described in this manuscript.

## Background

The persistence of HIV in long-lived latently-infected CD4+ T cells [1–5] continues to be a major barrier to a cure for HIV. Latently-infected CD4+ T cells are exceedingly rare in HIV-infected ART-suppressed individuals (about 1 per million cells [6]), and no methods currently exist to isolate or characterize these cells without ex vivo activation, which reverses latency. Multiple different mechanisms have been implicated in HIV latency [7, 8]. In peripheral blood mononuclear cells (PBMCs) and peripheral CD4+ T cells from HIV-infected ART-suppressed individuals, we recently showed that the major reversible blocks to HIV expression are blocks to HIV transcriptional elongation, distal transcription/polyadenylation (completion), and splicing [9, 10], while in rectal CD4+ T cells we observed blocks to initiation, completion, and splicing [10]. However, the cellular gene products mediating these blocks to HIV expression remain unclear. It is extremely challenging to investigate these questions using cells from ART-suppressed individuals because no methods exist to distinguish latently-infected cells from uninfected cells or cells infected with defective or non-inducible proviruses.

The development of HIV-infected cell lines, such as U1, ACH-2, J-Lat, and 8E5 [11–15] cells, has led to many fundamental discoveries and greatly advanced our understanding of HIV infection and the establishment of latency. These cell lines have been used in over 600 publications pertaining to HIV (PubMed results: [“U1 cell” and HIV] = 378; [“ACH-2 cell” or “ACH2 cell” and HIV] = 181; [“J-Lat cell” or “J-lat cell” and HIV] = 51; [“8E5 cell” and HIV] = 44), and they continue to be used in studies to investigate mechanisms of latent HIV infection, design or evaluate assays to measure the HIV reservoir [16], and test new therapies to reverse latency [17] or silence latently-infected cells. They differ in the parental cell of origin (U1: U937 pro-monocytic cells; ACH-2 and 8E5: A3.01 subclone of CEM T cells; J-Lat: Jurkat T cells), the replication capacity of the provirus (U1 and ACH-2 are replication-competent but harbor mutations in Tat and TAR, respectively [18, 19], while 8E5 and J-Lat cells are replication-defective), whether they produce viral particles at baseline (8E5) or only after activation (U1, ACH-2, J-Lat), and their responses to different activating stimuli and latency-reversing agents.

In vitro mechanistic studies using cell lines have yielded important insights and enabled research in settings where access to samples from HIV-infected individuals is limited or where ex vivo samples are unsuitable for large-scale assays requiring many cells. While multiple primary cell models of latency [20–27] have been developed to more closely mimic latency in vivo, it is not clear whether the mechanisms of latency in these primary cell models are the same as those in ART-suppressed individuals, and cell lines may have advantages in terms of their availability, ease of use, cost, frequency of infection, and known proviral sequence and integration site. Nonetheless, cell lines have inherent limitations that may reduce the degree to which they recapitulate latent HIV-1 infection and persistence in vivo. Some cell lines contain proviral mutations, such as the TAR mutation in ACH-2 or Tat mutation in U1 [18, 19], that contribute to the maintenance of their latent state. Transformed cell lines continuously proliferate, which render them fundamentally different from quiescent resting CD4+ T cells that are in G_0_ state [7]. Despite the clonal nature of their integration sites, the accumulation of cells with different proviral integration sites has been reported to occur in cell lines with replication-competent proviruses, such as ACH-2 and U1, over successive rounds of passaging [28].

Understanding the regulation of HIV latency at bulk and single-cell levels in tractable models of HIV latency, such as cell lines, can potentially provide insights into the cellular factors that may regulate HIV latency in vivo and may improve tools for screening new therapies designed to reactivate or silence latently-infected cells [17]. However, it is unclear how closely HIV latency in cell lines compares to cells from HIV-infected individuals. We hypothesized that the mechanisms underlying HIV latency differ between cell lines and cells from HIV-infected ART-suppressed individuals. To quantify the degree to which HIV expression is reversibly inhibited by mechanistic blocks at various stages of HIV transcription, we compared the bulk HIV transcription profile for seven latently-infected cell lines (U1, ACH2, and J-Lat 6.3, 8.4, 9.2, 15.4 and 5A8 clones; Table 1), three productively-infected cell lines (8E5, and reactivated J-Lat 9.2 and 5A8 clones), and cells from the blood of ART-suppressed individuals. We found that HIV-infected cell lines differed from each other and blood cells from ART-treated individuals in the blocks to HIV transcription underlying latency. To investigate the link between human and viral gene expression in individual cells, we also applied a novel panel of multiplex qPCR assays to quantify expression of seven HIV targets and 89 human genes in single cells from latently and productively HIV-infected cell lines. The expression of both HIV and cellular genes differed between cell lines, and we observed a surprising heterogeneity within some clonal cell lines in the single cell levels of polyadenylated HIV RNA, multiply-spliced HIV RNA, and certain cellular transcripts. However, some common features were shared across cell lines, such as impairments in cellular antiviral defenses, and deserve further study for their contribution to HIV persistence and latent infection in vivo.

**Table 1:** Cell lines analyzed in this study from NIH AIDS reagents program and the Greene laboratory

Cell line	Cell lineage	Parental cell line	Major integration sites [28, 97]	Virus	Replication competent virus	Viral mutation	Proviral copies reported per cell	Virus expression phenotype	References
U1	Monocyte	U937	AC079807.4 non-coding ORF (Chr.X)	HIV-1	Yes	Mutation in tat	2	Minimal constitutive virus expression Inducible with cytokines + PMA	[12]
ACH2	Lymphocyte	CEM	NT5C3A	LAV	Yes	Mutation in tar	1	Constitutively produces low levels of supernatant RT and p24 Infectious HIV-1 production inducible with PMA or TNFα	[13, 98]
J-lat 6.3	Lymphocyte	Jurkat	nd	HIV-R7/E-/GFP	No	Frameshift in env Nef defective	1	Minimal constitutive virus expression (secretion of incomplete viral particles) Inducible with PHA or PMA ± ionomycin	[15]
J-lat 8.4	Lymphocyte	Jurkat	FUBP1	HIV-R7/E-/GFP	No	Frameshift in env Nef defective	1	Minimal constitutive virus expression (secretion of incomplete viral particles) Inducible with PHA or PMA ± ionomycin	[15]
J-lat 9.2	Lymphocyte	Jurkat	PPP5C	HIV-R7/E-/GFP	No	Frameshift in env Nef defective	1	Minimal constitutive virus expression (secretion of incomplete viral particles) Inducible with PHA or PMA ± ionomycin	[15]
J-lat 15.4	Lymphocyte	Jurkat	UBA2	HIV-R7/E-/GFP	No	Frameshift in env Nef defective	1	Minimal constitutive virus expression (secretion of incomplete viral particles) Inducible with PHA or PMA ± Ionomycin	[15]
J-lat 5A8	Lymphocyte	Jurkat	MAT2a	HIV-R7/E-/GFP	No	Frameshift in env Nef defective	1	Minimal constitutive virus expression (secretion of incomplete viral particles) Inducible with T cell activation (α-CD3/CD28 antibodies), > 3.3 μg/ml PHA or PMA ± ionomycin	[97]
8E5	Lymphocyte	CEM	nd	LAV	No	Frameshift in pol	1	Constitutive expression of defective virus particles (no RT), with high p24 expression	[11, 99, 100]

_*nd* not determined_

## Results

### HIV DNA levels differ between cell lines

As a surrogate measure of HIV infection frequency in each cell line, we quantified HIV DNA levels using ddPCR assays for 5 different proviral regions and normalized them to cell numbers by DNA mass and by measured copies of the human gene Telomere Reverse Transcriptase (TERT) (Additional file 1: Fig. S1A, B). When normalized by DNA mass, U1 cells harbored an average of > 1 provirus per cell, as previously shown [12], and ACH-2, J-Lat 9.2 and J-Lat 5A8 harbored an average of 1 provirus per cell. In J-Lat 6.3 and J-Lat 8.4, we quantified 0.7 proviruses/cell; in J-Lat 15.4, 0.6 proviruses/cell; and in 8E5 cells, 0.5 proviruses/cell (Additional file 1: Fig. S1A). When normalized by TERT, the HIV DNA levels were lower, and all cell lines except U1 and J-Lat 5A8 showed an HIV infection frequency of < 1 provirus/cell (Additional file 1: Fig. S1B). These findings may reflect variability in HIV infection frequencies in cell lines and/or inaccuracies in the methods used to measure or normalize them in bulk cells, suggesting a role for single cell studies.

### HIV transcription profile differs between cell lines and cells from ART-suppressed individuals

**Fig. 1:**
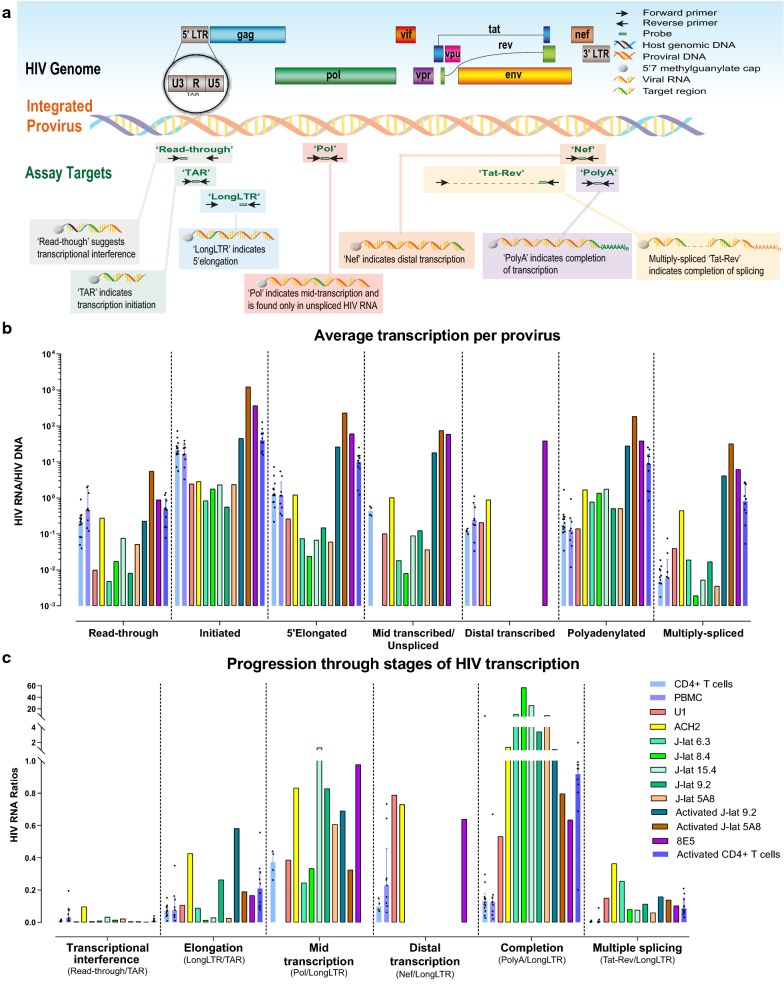
HIV genome and the targets for transcription profiling assays and bulk cell-associated HIV RNA levels. **a** This schematic shows the genetic organization of proviral HIV DNA and the HIV ‘transcription profiling’ assays targeting specific HIV RNA sequence regions suggesting transcriptional interference (Read-through) and progression through blocks to HIV transcriptional initiation (TAR), 5′ elongation (LongLTR), mid transcription (Pol), distal transcription (Nef), polyadenylation (PolyA), and multiple splicing (Tat-Rev). **b** HIV RNA levels normalized to HIV DNA (provirus) copies (ratio of each HIV RNA to LongLTR HIV DNA) (mean of 2 replicate measures of HIV RNA and DNA). **c** Ratio of one HIV RNA to another: Read-through normalized to TAR (transcriptional interference), LongLTR to TAR (elongation), Pol to LongLTR (mid transcription), Nef to LongLTR (distal transcription), PolyA to LongLTR (completion) and Tat-Rev to LongLTR (multiple splicing). For PBMCs, CD4+ T cells, and activated CD4+ T cells from HIV-infected ART-suppressed individuals (**b**, **c**), each individual is shown as a dot, the column height indicates the median, and bars represent 25–75%

To measure the degree to which different mechanisms inhibit HIV expression in each cell line, we measured the bulk cell levels of HIV transcripts that suggest transcriptional interference from neighboring cellular genes (“read-through” transcripts that inhibit initiation from the HIV promoter) and transcripts that indicate HIV transcriptional initiation (TAR), 5′ elongation (“LongLTR”), mid transcription (Pol; also indicates unspliced), distal transcription (Nef), polyadenylation (“PolyA”), and multiple-splicing (“Tat-Rev”) (Fig. 1a, Additional file 1: Fig. S1C). To correct for differences in the infection frequencies between cell lines and PBMCs or peripheral CD4+ T cells from HIV-infected ART-suppressed individuals, we calculated the average level of each transcript per provirus, as measured by the ratio of each HIV RNA region to levels of the R-U5-pre-Gag (LongLTR) HIV DNA region, which is present in one copy per provirus (Fig. 1b). Results were comparable when each HIV RNA was normalized to levels of the corresponding HIV DNA region measured using the same primers/probes (Additional file 1: Fig. S1D), suggesting that any differences between HIV RNA regions cannot readily be explained by proviral deletions or hypermutations in the primer/probe regions.

The unstimulated CD4 and PBMCs showed a continuous decrease from levels of initiated to 5′ elongated, mid-transcribed (Pol), distal transcribed (Nef), U3-polyadenylated, and multiply-spliced HIV transcripts, consistent with previously observed blocks to elongation, mid/distal transcription, polyadenylation, and splicing (Fig. 1b; Additional file 1: Fig. S1C, D). The latently-infected cell lines showed different patterns, with less of a decrease from initiated to polyadenylated transcripts, suggesting less block to elongation and completion.

HIV transcriptional initiation, as measured by TAR RNA/HIV DNA, tended to be lower in all latently-infected cell lines than unstimulated CD4 or PBMCs, higher in activated CD4+ T cells and activated J-Lat 9.2 cells, and even higher in the productively-infected 8E5 cells and activated J-Lat 5A8 cells (Fig. 1b). In contrast, levels per provirus of polyadenylated HIV transcripts tended to be higher in all latent cell lines compared to unstimulated cells from ART-suppressed individuals.

Levels per provirus of U3-R-U5 “Read-through” transcripts, which suggest transcriptional interference, were similar in ACH-2 cells and unstimulated CD4 or PBMCs, but the other latent cell lines tended to have lower levels. All unstimulated J-Lat clones and ACH-2 cells showed a surprising pattern where levels of initiated HIV transcripts were comparable to polyadenylated transcripts, suggesting little transcriptional block to elongation or completion, but paradoxically, polyadenylated transcripts were much greater than 5′ elongated (LongLTR) or mid transcribed (Pol) transcripts. We found no evidence of false positives in the assays for polyadenylated HIV transcripts or of inhibition in the assays for 5′ elongated or mid-transcribed (Pol) RNA or DNA. Moreover, levels of the “5′ elongated” and Pol HIV DNA were almost exactly the same and showed the expected 1:2 ratio to levels of the TAR and U3–U5 (read-through) HIV DNA regions (which are present at both ends of the provirus), suggesting that the differences in the HIV RNA are not explained by proviral mutations (Additional file 1: Fig. S1A, B). The surprising pattern of HIV RNAs in these J-Lat clones and ACH-2 cells suggests that many of their HIV transcripts result from upstream cellular run-on transcripts that continue into the proviral 5′ U3 region (upstream of the normal HIV transcription start site in the TAR sequence of the R region) but are polyadenylated after the R region of the 5′ LTR, prior to the U5 or Gag regions, so they are not detected by the U3–U5 “Read-through” assay. This data accords with prior findings in the J-Lat 9.2 clone, which described human/HIV hybrid transcripts that mostly terminated in the 5′ LTR [29]. Our data suggest all 5 J-Lat clones also have a form of transcriptional interference where most HIV transcripts are cellular run-on transcripts, but that they terminate prematurely because the transcription machinery recognizes the polyadenylation signal in the 5′ LTR.

Compared to unstimulated CD4 and PBMCs, U1 cells showed less of a decrease from 5′ elongated to distally transcribed and polyadenylated transcripts, suggesting less block to distal transcription and completion, while little or no decrease was observed in the ACH-2 cells and productively-infected cells. These data suggest that latently-infected cell lines differ from each other and from cells from ART-suppressed individuals in the HIV transcriptional blocks underlying latency (Additional file 2: Fig. S2).

### HIV latency in cell lines may be driven by transcriptional interference and/or blocks to elongation

To quantify the progression through various stages of (or blocks to) HIV transcription, we calculated the ratios of different HIV RNA regions (Fig. 1c and Additional file 3: Table S1). The ratio of U3-R-U5 Read-through to initiated (TAR) HIV transcripts was higher in ACH-2 cells (0.10) than the other cell lines or unstimulated CD4 or PBMCs, suggesting more transcriptional interference. In the J-Lat clones, the ratio of U3-R-U5 Read-through to initiated HIV transcripts was generally comparable to unstimulated CD4 or PBMCs. However, the large excess of initiated and polyadenylated over 5′ elongated or mid elongated transcripts in the J-Lat and ACH-2 cells suggests a different form of transcriptional interference in which cellular run-on transcripts get polyadenylated after the R region of the 5′ LTR.

The low ratios of 5′ elongated to initiated transcripts (LongLTR/TAR) in U1 cells (0.11) and most J-Lat clones were comparable to those in unstimulated CD4 or PBMCs, suggesting similarly low levels of elongation. In contrast, the ACH-2 cells showed a much higher ratio of 5′ elongated to initiated HIV transcripts (0.43), which was comparable to activated CD4 T cells and the productively-infected cell lines, suggesting little block to elongation. In all cell lines, levels of mid and distal HIV transcription (as measured by the ratio of Pol or Nef to LongLTR) were similar to or higher than the unstimulated CD4 or PBMCs. Likewise, the ratio of polyadenylated to 5′ elongated HIV transcripts was higher in all cell lines than in unstimulated CD4 or PBMCs, suggesting higher levels of transcriptional polyadenylation or “completion” in the latent cell lines (although in the case of the J-Lat clones and ACH-2, many of these are not full length).

While we have previously used the ratio of multiply-spliced to polyadenylated HIV RNA (Tat-Rev/PolyA) as a measure of splicing, the high levels of prematurely-terminated, polyadenylated transcripts in the J-Lat and ACH-2 cells confound interpretation of this ratio. The ratios of multiply-spliced to initiated and 5′ elongated transcripts were higher in all latent cell lines than in the unstimulated CD4/PBMCs (and sometimes comparable to activated CD4+ T cells), suggesting less baseline block to splicing. At the same time, J-Lat 9.2 and 5A8 cells showed an increase in these ratios with activation, suggesting some reversible block. These data suggest that the mechanisms that regulate HIV latency differ between latently-infected cell lines and cells from ART-suppressed individuals, with inhibition of HIV transcriptional initiation due to different forms of transcriptional interference in the ACH-2 and J-Lat cells, blocks to HIV-specific transcriptional initiation and elongation in U1 cells, and less baseline block to completion or splicing in all latent cell lines (Additional file 2: Fig. S2).

### Single cell multiplexed qPCR is sensitive and reproducible for detection of cellular and HIV transcripts

To determine whether there is cell-to-cell variability within cell lines, and to investigate cellular genes that may be associated with blocks to HIV expression, we developed a novel panel of assays to quantify multiple HIV targets and cellular transcripts at the single cell level using the Fluidigm C1 and Biomark HD platforms. Our 96-assay multiplex qPCR panel included five assays for different regions of HIV DNA or RNA (TAR, LongLTR, Gag, Pol, Nef), two assays specific for HIV RNA (for U3-polyadenylated [PolyA] and multiply-spliced [Tat-Rev] HIV transcripts), and 89 human cellular RNAs. Aside from a few housekeeping transcripts, the cellular genes (Additional file 4: Table S2) were all chosen based on prior studies suggesting their importance for HIV infection and/or latency. To simplify the analysis, we divided these cellular genes into 4 broad categories: (1) housekeeping transcripts; (2) markers of T cell phenotype and function (including markers of T cells and subsets, HIV coreceptors, markers of T cell activation and proliferation, negative T cell regulators such as PD-1, genes involved in apoptosis or survival, etc.); (3) cellular antiviral defenses, including restriction factors; and (4) genes implicated in human transcription/polyadenylation/splicing, HIV transcription, and HIV latency.

Validations were performed to determine the detection limit of each HIV assay, batch variation, correlation with bulk cell expression, and Biomark HD reproducibility. A multiply-spliced, polyadenylated HIV RNA standard and an HIV virion standard [9] were used to determine the sensitivity of our assays for HIV RNA in the single cell approach. As few as 5 copies of HIV RNA could be detected for all assays except PolyA, which was less efficient in this assay system than the bulk cell RT-ddPCR (Additional file 5: Fig. S3).

For the single cell analysis of viral and cellular gene expression, we studied 3 different latently-infected cell lines (U1, ACH-2, J-Lat 9.2) plus two different productively-infected cell lines (8E5, activated J-Lat 9.2). Because the bulk cell HIV transcription profile suggested very similar mechanisms of latency in all five J-Lat clones, we chose to study only one J-Lat clone at the single cell level; J-Lat 9.2 was chosen because it has been well-characterized with regard to the mechanism of latency (transcriptional interference). We did not include cells from ART-treated individuals because the expected frequency of HIV-infected cells (about 1 in 1000 circulating CD4+ T cells) is far too low expect capture of any HIV-infected cells with the C1 chips, which capture a maximum of 96 cells.

**Fig. 2:**
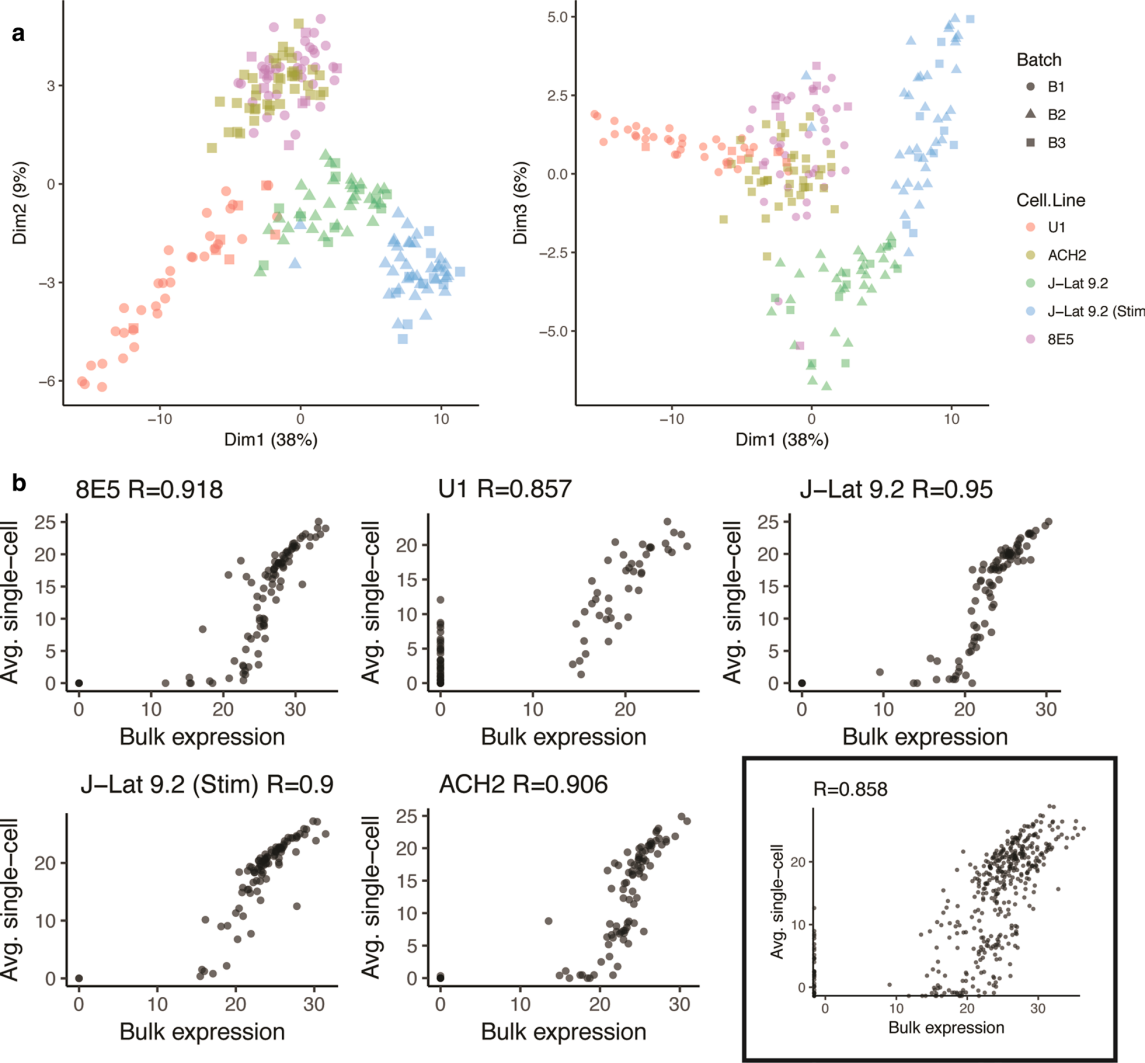
Single-cell multiplex qPCR analysis of HIV cell line models. **a** Principal component analysis of the gene expression levels (right plot: dimension 2 vs. dimension 1; left: dimension 3 vs. dimension 1). Single cells for each cell line are indicated by color and different shapes denote independent assays (batch). No batch effects were observed (P < 0.001, Wilcoxon rank sum test). **b** Scatter plots of average expression levels (40-cycle threshold; ~ log_2_) of all targets in single cells (y axis) vs. bulk cells (x axis) for each cell line and overall (box). High concordance was observed (R > 0.86 for all cell lines)

Cell viability was greater than 90% for all cell lines tested (median: 95%), except activated J-Lat 9.2 (median 86% viability prior to C1 integrated fluidic circuit loading). Each cell line was tested in at least two batches (Fig. 2a, Additional file 6: Fig. S4), and we selected cDNA from individual cells (11 J-Lat 9.2 and 3 U1 cells) for repeat-testing in two independent Biomark experiments (Additional file 6: Fig. S4). A total of 40 unique U1 cells, 40 ACH-2, 44 8E5, 41 unstimulated J-Lat 9.2, and 45 activated J-Lat 9.2 were analyzed (Additional file 7: Table S3). At least one HIV target was detected in 40/40 U1, 40/40 ACH-2, 43/44 8E5, 40/41 unstimulated J-Lat 9.2, and 45/45 activated J-Lat 9.2 cells. For each cell line, the average single cell expression of all targets correlated with bulk cell expression (R ≥ 0.86; Fig. 2b). With repeat testing of the cDNA from individual cells, levels of HIV and cellular targets were highly reproducible from one Biomark run to the next (Additional file 6: Fig. S4A), and the “drop out” frequency was relatively low, even for low expression targets (Additional file 6: Fig. S4B). Batch variation was formally assessed using the ComBat method [30], but this did not appreciably alter the analysis (tSNE plot; Additional file 8: Fig. S5), so further analyses were conducted without batch correction adjustment. These validations indicate that the gene expression analyses using the Biomark system were highly sensitive and reproducible.

### Host gene expression accounts for most of the difference between cell lines

We hypothesized that differences between cell lines would reflect the degree of HIV expression (latent vs. productive), especially because many of the cellular genes in our panel were selected for their reported associations with HIV transcription and latency. Contrary to our expectations, the tSNE and PCA plots revealed clustering driven primarily by host gene expression. U1 cells, which are derived from a monocytic cell line [12], formed a distinct cluster in the tSNE plot (Additional file 8: Fig. S5). ACH-2 and 8E5 cells, which are both derived from a human T cell line originating from acute lymphoblastic leukemia [11, 13, 14, 31, 32], clustered together in both the PCA and tSNE plots despite the fact that one is latently-infected and the other is productively-infected (Fig. 2a, Additional file 8: Fig. S5). Furthermore, both untreated and activated J-Lat 9.2 cells clustered in close proximity to one another despite disparities in expression of both HIV and cellular targets (Fig. 2a, Additional file 8: Fig. S5).

In the principal component analysis, a total of 46.9% of the variation was driven by dimension 1 (which distinguished U1, ACH-2/8E5, and J-Lat cells) and dimension 2 (which distinguished ACH-2/8E5 from the others), which were both driven by clusters of host genes (Fig. 2a, Additional file 9: Fig. S6). HIV Nef was included in a cluster with CD4, CD44, and interferon α (IFNA1; IFNα), but the role of Nef was confounded by the fact that this HIV region is deleted in J-Lat cells but not the other cell lines (Fig. 2a, Additional file 9: Fig. S6). Dimension 3 (which distinguished unstimulated J-Lat from the others) was driven by all the other HIV targets (including TAR, LongLTR, Gag, Pol, PolyA, and Tat-Rev), but accounted for only 5.9% of the variation observed. Taken together, these data suggest that most of the observed difference between latently- and productively-infected cell lines is driven by host gene expression rather than HIV expression.

### Single cell analysis reveals cell-to-cell variation in HIV target levels

**Fig. 3:**
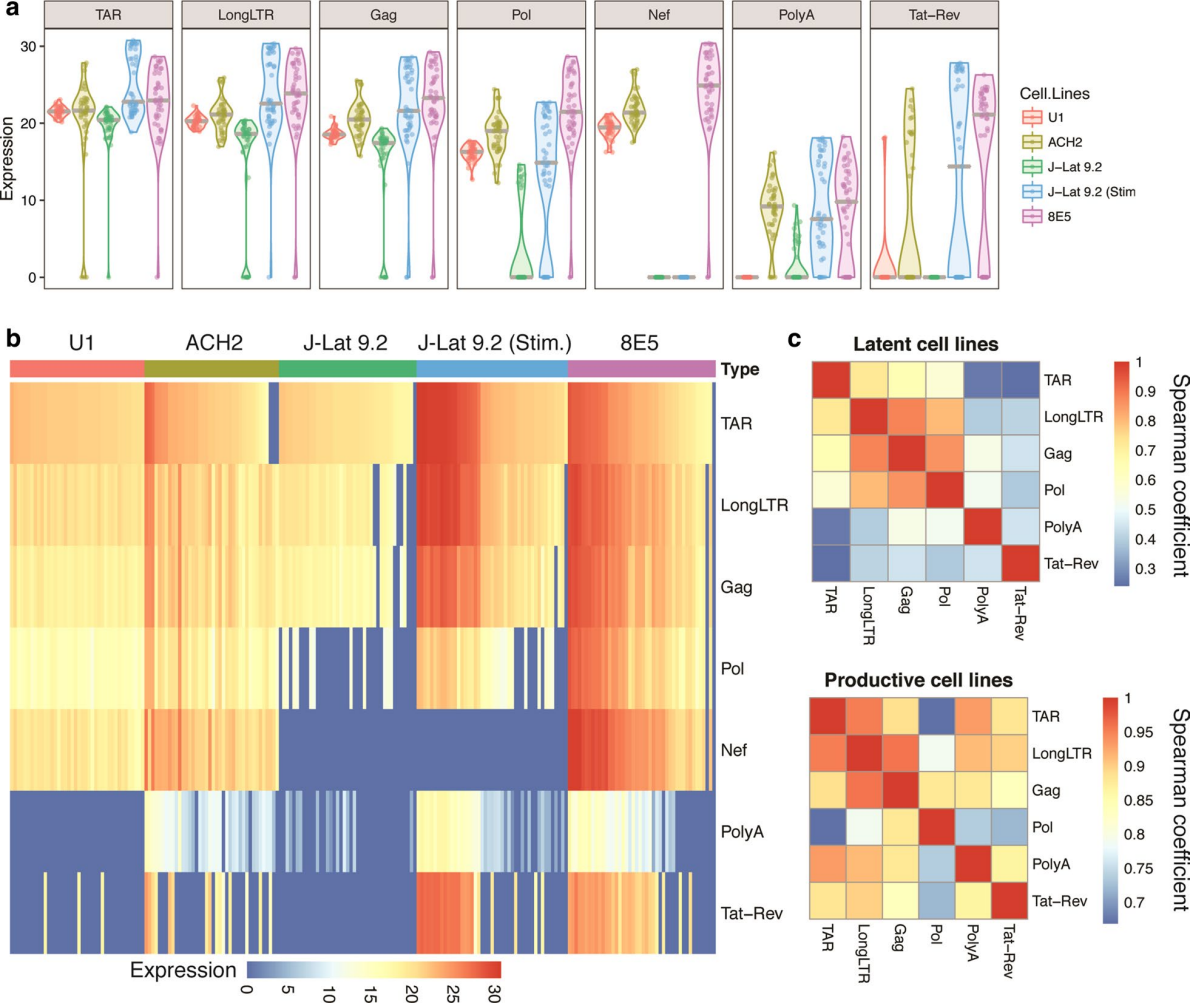
Single cell differences in HIV target levels. **a** Violin plots representing spread and variability of HIV target levels (40-cycle threshold). Individual cells are denoted by dots and median levels are indicated by horizontal lines. **b** Heatmap of cell-to-cell variation in levels of each HIV target (rows). Cells are grouped on the basis of cell line and ordered from high to low level of HIV TAR. Each vertical line represents a single cell. Color scale (below) indicates HIV target levels. **c** Correlation matrices for levels of each HIV target in latently-infected (U1, ACH-2, J-Lat 9.2; upper panel) and productively-infected (activated J-Lat 9.2 and 8E5; lower panel) cell lines. Nef has been excluded from these analyses. Color scale denotes Spearman coefficients

To determine whether there is single cell variation in HIV infection or transcription within cell lines, we measured levels of HIV TAR, LongLTR, Gag, Pol, Nef, PolyA and Tat-Rev at the single cell level and visualized the range in levels across all cells using violin plots (Fig. 3a) and heat maps (Fig. 3b). The average HIV expression in single cells tended to mirror our observations in bulk cell analyses (Fig. 1b), with several important distinctions. Despite the sensitivity of our Tat-Rev assay (Additional file 5: Fig. S3), we detected Tat-Rev in none of the 41 untreated J-Lat 9.2 cells and only 3/40 U1 cells, consistent with our bulk studies that estimate a frequency of < 0.1 copies per provirus (Fig. 1b). Tat-Rev was detected in 15/40 ACH-2 cells, 30/44 8E5 cells, and 23/45 activated J-Lat 9.2 cells (Fig. 3b); all 3 cell lines showed a bimodal expression that is masked in bulk cell analyses. The PolyA assay performed less efficiently than the other HIV assays in the Biomark system (Additional file 5: Fig. S3), so the lower levels of these transcripts at the single cell level do not reflect our observations in bulk cells using RT-ddPCR.

As expected, the levels of specific HIV targets varied considerably between cell lines designated ‘latently’ and ‘productively’ infected (Fig. 3a, b). Of the three latently-infected cell lines (ACH-2, J-Lat 9.2 and U1), J-Lat 9.2 tended to exhibit the lowest levels of all HIV targets except PolyA, which was detected more frequently in J-Lat 9.2 than in U1 cells (note that Nef is not present in the full-length integrated HIV construct of J-Lat cells [15]). This finding was consistent with our observations in bulk cells (Fig. 1b). U1 and ACH-2 cells showed comparable levels of HIV TAR, while ACH-2 cells showed higher median levels of the other HIV targets and a higher frequency of Tat-Rev+ cells. U1 cells also tended to exhibit less variation in single cell HIV levels (Fig. 3a, b). U1 and J-Lat 9.2 cells exhibited the lowest levels of HIV expression, which accords with their ‘latent’ phenotype and may more closely model levels observed in ex vivo CD4+ T cells from HIV-infected individuals.

On activation of J-Lat 9.2, we observed marked upregulation of all HIV targets, including polyadenylated (PolyA) and multiply-spliced (Tat-Rev) HIV transcripts (Fig. 3a, b). Activated J-Lat 9.2 and productively-infected 8E5 cells demonstrated higher median levels of all HIV targets when compared to the three latently-infected cell lines. Both activated J-Lat 9.2 and 8E5 cells also showed cell-to-cell variability in all HIV targets, with bimodal expression of polyadenylated and multiply-spliced HIV transcripts. These results help confirm the differences between cell lines that were observed through the bulk cell measurement of HIV transcript levels but also reveal a surprising heterogeneity in single cell levels of polyadenylated and multiply-spliced HIV transcripts within the clonal populations of ACH-2, 8E5, and activated J-Lat cells.

### Single cell HIV levels suggest blocks to HIV transcription in latent cell lines

Within individual cells, we observed differences in levels of HIV regions, consistent with previously observed blocks to HIV transcription (Fig. 1). In cell lines designated as ‘latent’ (ACH-2, J-Lat 9.2, and U1), correlations tended to be stronger between adjacent HIV regions and weaken with distance along the proviral genome (Fig. 3c; the correlations between Nef and other transcripts are confounded by the lack of Nef in the integrated provirus in J-Lat 9.2 cells and were not included in these analyses). In the latent cell lines, 5′ regions (TAR, LongLTR, Gag, and Pol) correlated strongly with each other but weakly with PolyA and Tat-Rev, which could reflect transcriptional blocks and/or the fact that the latter two assays are RNA-specific. In contrast, the productive cell lines showed greater correlations between PolyA or Tat-Rev and the 5′ targets (TAR, LongLTR, Gag) (Fig. 3c). Moreover, the correlations between most HIV transcripts were significantly greater in the productive cell lines (Spearman coefficient > 0.8 for all correlations except Pol), suggesting that existing blocks to transcription were reversed on stimulation (J-Lat) or that minimal blocks to transcription operate in productively-infected cells (8E5).

### HIV-infected cell lines vary in T cell phenotypic markers and some genes implicated in latency

**Fig. 4:**
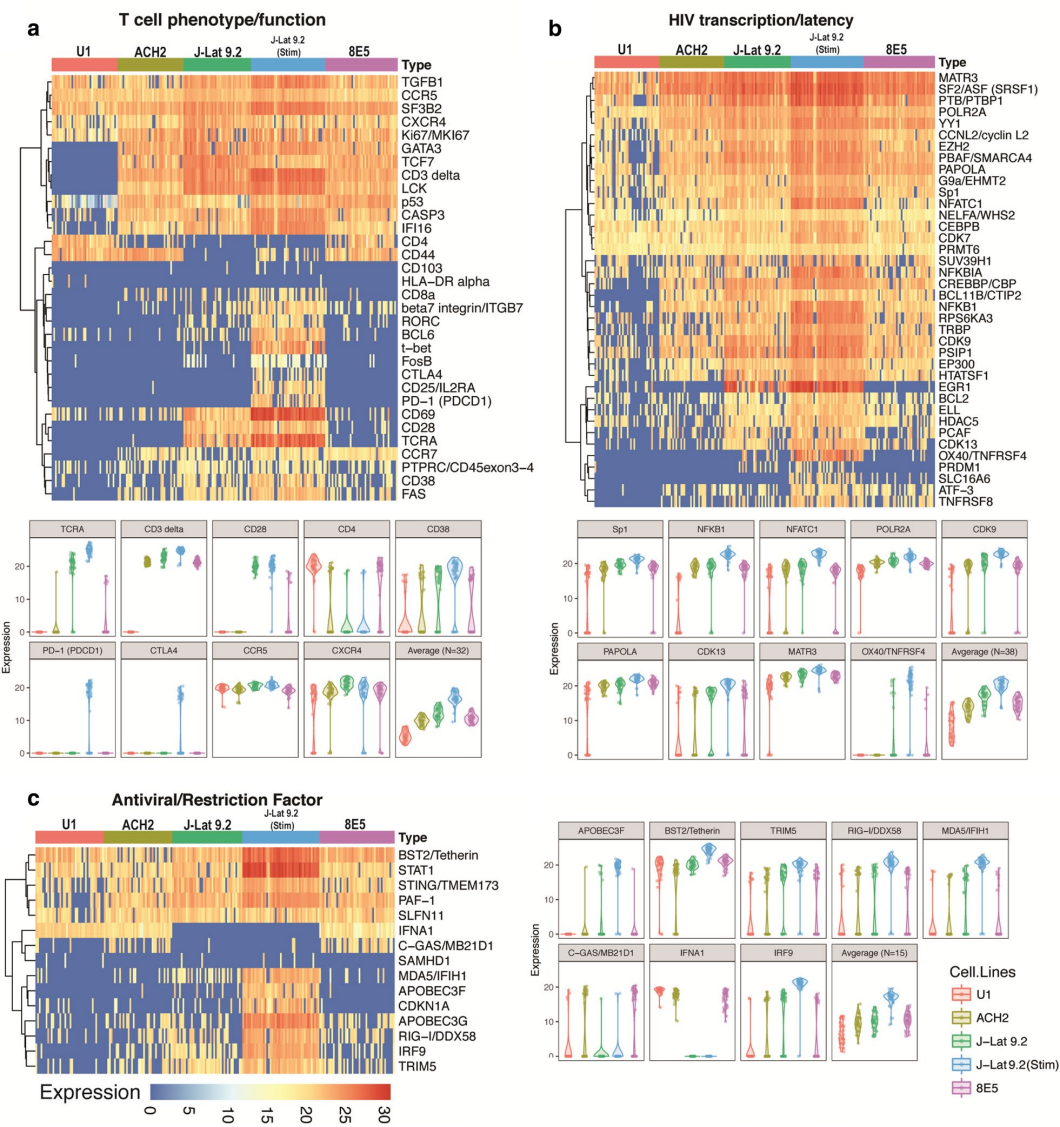
Single cell variation in cellular gene expression. Single cell RNA levels are shown for cellular genes associated with **a** T cell phenotype and function; **b** HIV transcription and latency; and **c** antiviral and restriction factors. Cells are grouped by cell line and ordered from high to low level of HIV TAR. Each vertical line represents a single cell. Rows indicate different genes. Color scale (below) denotes expression level (40-C_T_). Dendrograms of unsupervised clustering are indicated to the left of heat map. Heatmaps illustrate cell-to-cell variation in cellular gene expression. Violin plots are shown for selected host genes in each category, with individual cells denoted by dots

We hypothesized that specific cellular factors are likely to be associated with differential HIV transcription in HIV-infected cell lines. Accordingly, we performed hierarchical clustering (by HIV TAR level) of our gene expression data for T cell phenotypic markers (Fig. 4a), factors reportedly associated with HIV transcription or latency (Fig. 4b), and cellular antiviral or restriction factors (Fig. 4c). The expression of housekeeping genes (GAPDH, RPL13a and PPIA) was high across all cell lines (Additional file 10: Fig. S7). Strikingly, J-Lat 9.2 cells expressed little or no CD4 even though they expressed high levels of T cell receptor alpha chain (TCRA), CD3 delta (CD3), and CD28 (Fig. 4a). In contrast, ACH-2 and 8E5 cells expressed CD3 and low, bimodal expression of CD4 but little or no TCRA or CD28. Overall, T cell phenotypic and functional markers were expressed at similar average levels in ACH-2 and 8E5 despite their divergent levels of HIV targets. As expected, monocyte-derived U1 cells expressed CD4 but not the T cell phenotype/function-associated targets in our panel, including TCRA, CD3 delta, CD28, CTLA-4, transcription factor 7 (TCF7), and nonreceptor protein-tyrosine kinase p56 (LCK) (Fig. 4a). Of the 5 cell types, only the activated J-Lat cells expressed programmed cell death protein 1 (PD-1) and CTLA-4 transcripts (Fig. 4a).

We also observed considerable cell-to-cell variation in expression of some genes reportedly associated with HIV transcription or latency, including histone methyl transferase SUV39H1 in U1 cells, and TNF Receptor Superfamily member 8 (TNFRSF8, CD30) in J-Lat 9.2, ACH-2, and 8E5 cells (Fig. 4b). Nonetheless, J-Lat 9.2, ACH-2, and 8E5 expressed many of the genes in our panel that are reportedly associated with HIV transcription and/or latency (Fig. 4b, Additional file 10: Fig. S7). In contrast, U1 exhibited limited expression of many of these genes (zinc-finger transcription factor BCL11B, TNF Receptor Superfamily Member 4 [TNFRSF4, OX40, CD134], PR domain zinc finger protein 1 [PRDM1, Blimp-1], ATF-3, and TNFRSF8 [CD30]) and exhibited generalized low RNA expression. Compared to the latent cell lines and even 8E5, activated J-Lat 9.2 exhibited higher average expression levels of most genes implicated in HIV transcription or latency (Fig. 4a, b). These data underscore similarities and differences between cell lines in expression of some genes that have been implicated in HIV latency, as well as heterogeneity within clonal cell lines in expression of some of these genes.

### HIV-infected cells lines may have impairments in antiviral defenses

To investigate whether downregulation of cellular antiviral defenses could contribute to persistent HIV-infection or latency in cell lines, we measured single cell expression of cellular antiviral genes, including restriction factors. The expression of constitutive apolipoprotein B mRNA-editing catalytic polypeptide-like (APOBEC) variants APOBEC3G and APOBEC3F differed among the cell lines, with little or no expression of APOBEC3G (U1) and APOBEC3F (U1, ACH-2, and 8E5) to high expression levels in activated J-Lat 9.2 (Fig. 4c). IFNα was not detected in J-Lat 9.2 cells irrespective of activation state, while U1, ACH-2, and 8E5 expressed IFNα but often did not express Interferon Response Factor 9 (IRF 9) (Fig. 4c). However, IRF9, Bone marrow Stromal antigen 2 (BST2, CD317, tetherin), and Signal Transducer and Activator of Transcription 1 (STAT1) were strongly upregulated with activation of J-Lat 9.2 cells (Fig. 4c). All cell lines tested demonstrated little or no expression of SAMHD1 (Fig. 4c), which is highly expressed in resting CD4+ T cells and reportedly suppresses HIV-1 LTR-driven gene expression [33].

We also measured single cell expression of antiviral pattern recognition receptors (PRR), including cytoplasmic viral RNA sensors (Retinoic acid-Inducible Gene 1 [RIG-I], Melanoma Differentiation-Associated protein 5 [MDA5]) and viral DNA sensors (Cyclic GMP-AMP Synthase [C-GAS], Interferon gamma Inducible Protein 16 [IFI16]) (Fig. 4c). Expression of RIG-I was variable among the cell lines tested, ranging from low expression in U1 to strong upregulation in activated J-Lat 9.2 (Fig. 4c). Similarly, MDA5 levels were low in U1, ACH-2 and 8E5 but comparatively higher in J-Lat 9.2, particularly with activation (Fig. 4c).

On the other hand, J-Lat 9.2 cells expressed very little C-GAS, even upon activation (Fig. 4c), although most J-Lat 9.2 cells expressed IFI16 (Fig. 4a; Additional file 10: Fig. S7), the levels of which were increased on activation. U1 cells also expressed very little C-GAS, in contrast to both ACH-2 and 8E5, in which C-GAS expression was comparatively higher (Fig. 4c). The most genetically-similar cell lines, ACH-2 and 8E5, tended to express the same antiviral factors, with no evidence to suggest that increased HIV expression (as observed in 8E5) results in modulation of these factors at the transcriptional level. Interestingly, low/no expression of different antiviral factors was observed in each cell line, suggesting that inherently attenuated antiviral responses contribute to persistent HIV infection and/or latency in these cell lines.

### HIV RNA expression correlates with host gene expression in J-Lat cells

**Fig. 5:**
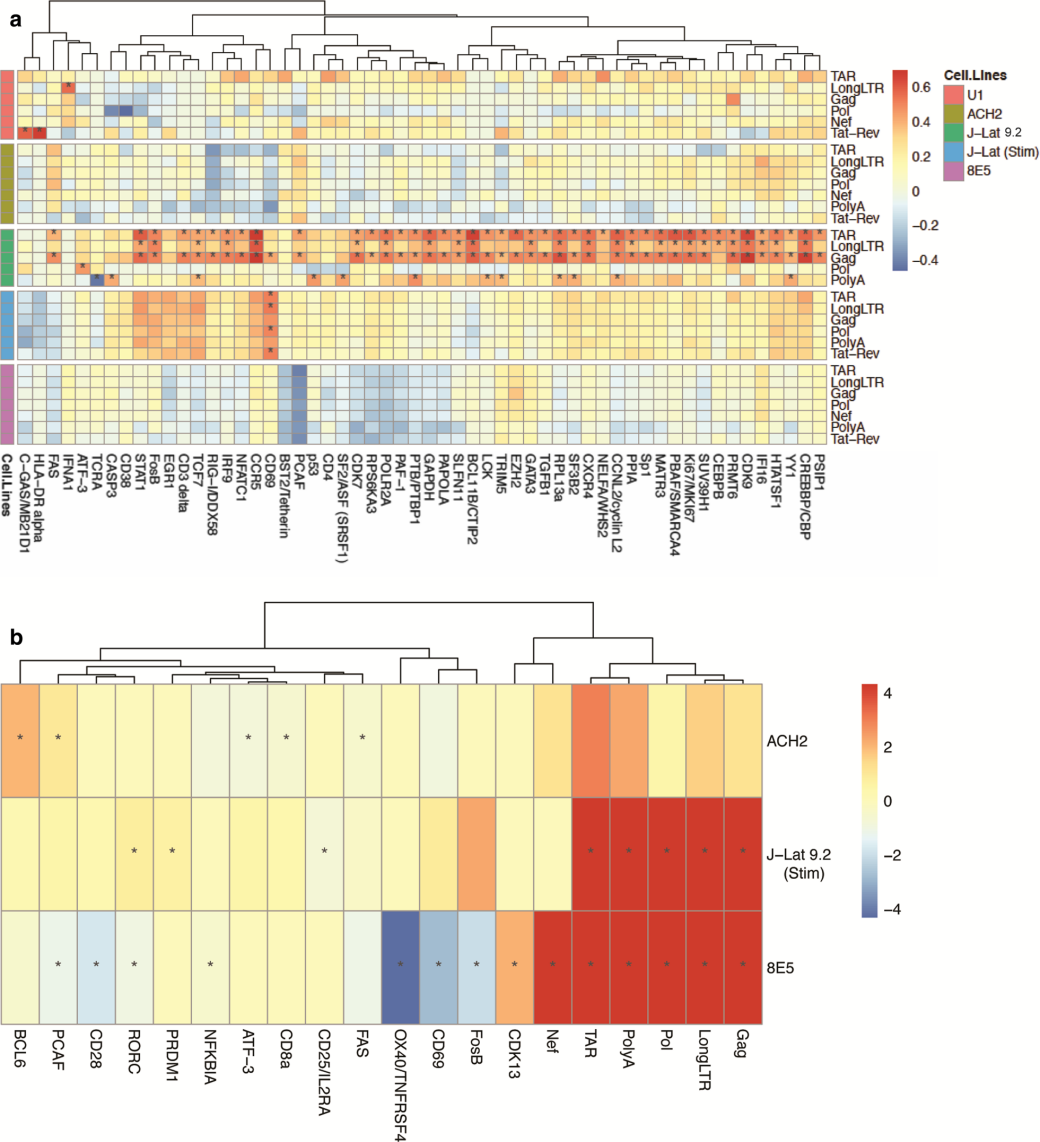
Correlation between HIV targets and cellular transcripts. **a** Correlation matrix showing degree of correlation between expression of each cellular gene (columns) and each HIV target (rows). HIV targets are grouped by cell line. Color scale (right) indicates Spearman r values. **b** Differential expression of cellular genes between Tat-Rev+ and Tat-Rev− subpopulations of ACH-2, activated J-Lat, and 8E5 cells. Color scale denotes difference in expression level (log_2_) in Tat-Rev+ cells. P values were calculated using the Wilcoxon rank sum test and corrected for false discovery rate (FDR) using the Benjamini–Hochberg correction; asterisks (*) denote FDR-adjusted P values < 0.05. Dendrograms (above) show unsupervised clustering of cellular factors. Cellular genes for which no expression was detected were excluded from these analyses

The cellular genes in our panel were selected for their prior reported associations with HIV infection and latency. We hypothesized that expression of specific cellular factors associated with HIV transcription/latency or antiviral responses might correlate with the levels of HIV targets across different cell lines. To address this question, we measured the correlation between expression of each cellular gene and each HIV target in each cell line (Fig. 5a). In U1, ACH-2 and 8E5, no significant correlations were observed (after correction for multiple comparisons) except for those driven by the low detection of targets (Additional file 11: Fig. S8).

In unstimulated J-Lat 9.2, however, we observed many cellular genes for which expression positively correlated with HIV targets, and this correlation was also observed with housekeeping genes, suggesting that HIV transcription in unstimulated J-Lat 9.2 cells reflects the degree of human cellular transcription (Fig. 5a). Positive correlations with higher correlation coefficient values and FDR < 0.05 included: CCR5, BCL11B, cyclin-dependent kinase 9 (CDK9), CREB Binding Protein (CREBBP), and STAT1 (positively correlated with TAR, LongLTR, and Gag; Fig. 5a and Additional file 12: Fig. S9). In activated J-Lat 9.2 cells, the correlation coefficient values were lower for all but a small subset of human genes (including STAT1, FOSB, EGR1, CD3, TCF7, CCR5, and CD69), and FDR < 0.05 was observed only with CD69, which correlated positively with multiple HIV targets, including Tat-Rev (Fig. 5a; Additional file 12: Fig. S9). CD69 increases transiently after T cell activation but has also been described as a marker for tissue resident memory T cells. Surprisingly, no significant correlations were observed between the HIV targets and other putative markers of T cell activation (including CD25, HLA-DR, CD38) in the activated J-Lat 9.2 cells, suggesting that the heterogeneity in HIV expression in these cells may not simply reflect differences in the degree of activation. Moreover, the cellular factors that correlated with HIV expression in unstimulated or activated J-Lat 9.2 cells (such as CD69) showed no correlation or even trends towards opposite correlations with HIV targets in the other cell lines. The lack of a consistent correlations across all cell lines could reflect differences in the genetic backgrounds of the various cell lines or differences in the mechanisms that maintain latency in each cell line.

To further delineate differential expression mediated by activation, we compared expression of all targets between untreated and activated J-Lat 9.2 (Additional file 13: Fig. S10). In addition to increased expression of HIV targets on activation, we found upregulation of multiple phenotype/function associated genes, including T-box transcription factor (t-bet), B Cell Lymphoma 6 (BCL6), CD44, TCRA and CD69, of which the most marked fold change was observed in t-bet expression (Additional file 13: Fig. S10). Multiple antiviral factors, including APOBEC3G, Cyclin-dependent Kinase Inhibitor 1A (CDKN1A), RIG-I, MDA5, Interferon Regulatory Factor (IRF9), and STAT1, also increased in expression on activation (Additional file 13: Fig. S10). Notably, increased HIV expression also coincided with increased expression of OX40, a cell survival associated receptor, and NFΚBIA, which plays a role in mediating the transcriptional activity of NFΚB (Additional file 13: Fig. S10). Two of the most differentially expressed genes, OX40 and CDKN1A, both play anti-apoptotic roles. Although activation of the J-Lat 9.2 cells increased expression of both HIV and cell survival associated factors, it is not clear whether these are independent effects of activation or whether there is a link between HIV expression and cellular factors involved in apoptosis/survival.

### Tat-Rev+ cells show differential expression of genes associated with activation, transcription, and cell survival

Multiply-spliced Tat-Rev RNA has been proposed as a marker for productive infection, but we found single cell variation in Tat-Rev RNA even in the activated or productively-infected cells. To further investigate cellular factors that may be associated with HIV splicing and/or productive infection, we next determined how Tat-Rev+ and Tat-Rev− cells within each cell line differ in the other HIV targets and expression of human genes. In activated J-Lat 9.2 and 8E5, the Tat-Rev+ cells exhibited higher levels of HIV TAR, LongLTR, Gag, Pol, and Poly A (*FDR *< 0.05; Fig. 5b), reaffirming our previous correlations that suggest higher expression of other HIV targets occurs in cells that also express Tat-Rev (Fig. 3c). Tat-Rev+ and Tat-Rev− cells differed in their expression of cellular factors involved in activation (CD25, CD28, CD69), tissue retention (CD69), transcription (BCL6, FosB, ATF3, RORC, and P300/CBP-Associated Factor [PCAF]), and apoptosis/survival [TNFRSF4/OX40, Fas], but the specific genes varied between cell lines and sometimes showed opposite expression in different cell lines (Fig. 5b). Within the activated J-Lat 9.2 cells, Tat-Rev+ cells showed less expression of the early activation marker CD25 (despite a trend towards more CD69), while within the 8E5 cells, Tat-Rev+ cells showed lower expression of CD69 and the T cell coreceptor CD28. These findings suggest that the differences in HIV expression are not entirely driven by differences in T cell activation. Tat-Rev+ 8E5 cells showed lower levels of the transcription regulators FosB and PCAF, while Tat-Rev+ ACH-2 cells showed higher levels of BCL6 and PCAF. Tat-Rev+ ACH-2 cells showed lower expression of the pro-apoptotic factor Fas, while Tat-Rev+ 8E5 cells had lower levels of the survival factor OX40/TNFSR4. Taken together, these data suggest a complex relationship between productive HIV infection and genes involved in activation, transcription, and apoptosis/survival.

## Discussion

This study provides an in-depth analysis of HIV transcript profiles and cellular gene expression in commonly-studied HIV-infected cell lines. We found that all cell lines differ from each other and from blood cells from HIV-infected ART-suppressed individuals in the HIV transcriptional blocks underlying latency. We observed marked variation in HIV and cellular gene expression both between and within cell lines, which has not been previously characterized, but we also identified several features that were common across multiple cell lines, such as low expression of cellular antiviral factors.

U1 cells showed a strong block to HIV transcriptional elongation, which is consistent with previous studies showing that these cells harbor a mutation in Tat and suboptimal levels of Tat protein [34]. U1 cells may also have a block to HIV transcriptional initiation, since HIV transcriptional initiation was lower than in cells from ART-suppressed individuals and prior studies have also showed alterations in the nuclear factor-kappa B (NF-κB) moieties [35]. However, we did not measure the extent to which these blocks can be reversed by monocyte-activating stimuli. It is also worth noting that U1 cells harbor an average of 2 proviruses per cell, so it is possible that different mechanisms inhibit expression of the two proviruses.

Prior publications have suggested that ACH-2 cells also have a block to elongation, which has been attributed to a mutation in the TAR region (C^37^ → T) [18]. In contrast, we observed a high ratio of elongated to initiated (TAR) transcripts in the ACH-2 cells, suggesting little block to elongation, though it was possible that our measures of TAR RNA were not accurate despite correcting our probe sequence for the mutation. The ratio of read-through (U3-R-U5) to initiated transcripts was higher in the ACH-2 cells than other cell lines or most ART-suppressed individuals, suggesting that transcriptional interference operates to a greater degree in this cell line. Transcriptional interference has been described in J-Lat clones [29], but to our knowledge, this is the first time it has been shown in ACH-2 cells.

In all the J-Lat clones studied, we observed a surprising pattern where levels of initiated (TAR) and polyadenylated (U3-polyA) HIV transcripts were much greater than 5′ elongated (LongLTR) or mid-transcribed (Pol) transcripts. The best explanation for this pattern is that most of these transcripts are hybrid human/HIV run-on transcripts that contain the 5′ U3 region and the TAR loop but become prematurely terminated and polyadenylated in the 5′ LTR. Other explanations are possible, but seem less likely. The measured levels of polyadenylated transcripts could be falsely high, but prior validations have shown similar efficiencies for all the HIV RNA assays [9], with rare false positives that are generally limited to one droplet, while the J-Lats showed many PolyA+ droplets at the expected amplitude and the negative controls remained negative. Moreover, it seems too much of a coincidence that levels of polyadenylated and initiated transcripts are almost exactly equal, and that this is true for all the J-Lat clones but none of the other cell lines. It is also possible that both the assays for 5′ elongated and Pol transcripts gave falsely low results. However, we did not see any evidence for inhibition in the ddPCR plots for either the 5′ elongated or Pol regions in either the DNA or RNA, and we found no evidence for proviral mutations affecting these regions (Additional file 1: Fig. S1A, B). Moreover, it seems unlikely that there would be inhibition in the 5′ elongated and Pol assays but not the PolyA assay, since all derive from aliquots of the same reverse transcriptase reaction, or that there would be inhibition in all of the J-lat clones and none of the other cell lines. A third possibility is some form of alternative splicing. Pol is removed by the first splicing event and this could contribute to lower levels of Pol, but almost all studies show a vast excess of unspliced over spliced HIV RNA, and the 5′ elongated region is proximal to any known splice donor site and should not be reduced by splicing.

The surprising HIV transcription profile in the J-Lats is best explained by read-through transcripts that are polyadenylated after the R region of the 5′ LTR, suggesting a type of transcriptional interference that also prevents elongation. Additional evidence comes from a previously published manuscript by Lenasi et al., where the authors reported the presence of human/HIV hybrid (read-through) transcripts in J-Lat 9.2 cells and determined where these transcripts terminate by designing a panel of RT-PCR assays where the forward primer is in the cellular integration site and the reverse primers target different regions of the proviral genome [29]. They found that most of these read-through transcripts were detected by a reverse primer in the R region of the LTR (before the polyadenylation site) but not by reverse primers beyond the 5′ LTR, indicating that most of them terminate somewhere in the distal 5′ LTR [29]. These data corroborate the pattern we observed in the J-Lat cells as well as our explanation that most of their read-through transcripts get polyadenylated in the 5′ LTR. At the same time, the strategy used by Lenasi et al. did not enable them to determine whether these read-through transcripts become polyadenylated and why transcription stops in the 5′ LTR, while our data provide an explanation: in these J-Lat clones, the host transcription machinery that produces these read-through transcripts often recognizes the viral polyadenylation signal that is present in the 5′ LTR, leading to termination and polyadenylation.

The pattern of read-through, prematurely-terminated, polyadenylated transcripts observed in the J-Lat clones was also observed in the ACH-2 cells and CD4+ T cells from one HIV-infected participant, but was very different from the pattern found in U1 cells or most ART-suppressed individuals. However, in the cell lines studied here as well as all ART-suppressed individuals, we also found canonical U3-R-U5 “read through” transcripts, suggesting that some cellular run-on transcripts may continue into the U5 region and possibly beyond the 5′ LTR. In the J-Lat 9.2 cells, activation increased all HIV transcripts and there was much less difference between TAR, LongLTR, Pol, and PolyA, suggesting most of these transcripts no longer terminate in the 5′ LTR.

Since transcriptional interference is due to run-on transcription from neighboring host genes, the presence and degree of transcriptional interference are likely dependent on the proviral integration site, and transcriptional interference would not be expected in cell lines or primary cells where the provirus has integrated into non-transcribed regions. The proviral integration site and its chromatin environment likely play a role in transcriptional silencing of HIV-1 [15, 36, 37]. Based on their studies in J-Lat clone A1 (not studied here), in which the provirus is integrated into the X chromosome, Dieudonné et al. have proposed that HIV latency is associated with the location of the provirus in the nucleus (close to the nuclear membrane), and that additional suppression of HIV transcription can be caused by spatial proximity of the provirus to heterochromatic regions (such as the centromere of neighboring chromosome 12) [37]. Consequently, proviral integration in or proximity to non-transcribed regions, such as centromeres and telomeres, could also contribute to transcriptional silencing/latency in other cell lines or primary cells.

The ratios of polyadenylated to 5′ elongated HIV transcripts were higher in all latent cell lines compared to ART-suppressed individuals, suggesting more polyadenylation (less block to “completion”), although in the J-Lat cells, many of these “completed” transcripts are likely not full length. As measured by the ratio of Tat-Rev/TAR and Tat-Rev/LongLTR, all latent cell lines had higher levels of multiple splicing than in the unstimulated cells from ART-suppressed individuals, and these ratios were sometimes comparable to activated cells from ART-suppressed individuals. However, an important caveat that could affect these ratios is that the Tat-Rev region is frequently deleted in proviruses from ART-treated individuals [38]. Nonetheless, these data suggest that a block to splicing contributes less to latency in these cell lines. In terms of the levels of HIV transcriptional initiation, block to elongation, and slight block to distal transcription/completion, the U1 cell line appeared most similar to PBMCs and CD4+ T cells from ART-suppressed individuals, even though it is a monocytic cell line. Productively-infected (8E5, activated J-Lat 9.2 and 5A8) cells showed little block to HIV transcriptional elongation or completion and less block to splicing, suggesting this is the pattern of productive infection.

In accord with our bulk analysis, the single-cell levels of HIV transcripts varied depending on whether a cell line was latently-infected or productively-infected. Within a given cell line, marked differences were observed in HIV transcript and cellular gene expression at the single cell level. The cell-to-cell variability in HIV expression was lower for the latent cells (especially U1) and greater for the productive cells, particularly for polyadenylated and especially multiply-spliced transcripts. In terms of the levels (in both bulk and single cell assays) and cellular distribution of all HIV transcripts except TAR, the ACH-2 cells appear to be intermediate between the other latent cell lines and the productive cell lines. The ACH-2, activated J-Lat, and 8E5 cells all appeared to show a bimodal distribution of Tat-Rev. It is possible that these latter three cell types contain a mixture of latently- and productively-infected cells or a continuum, either because of stochastic, noise-driven differences in HIV transcription or variable expression of the cellular genes governing HIV transcription and splicing [39, 40]. It is also possible that the activation did not exert uniform effects on the J-Lat 9.2 cells (Fig. 3b). A similar variability in HIV expression and/or response to activation may operate in vivo, since prior studies using primary cell latency models and cells from ART-suppressed individuals have shown that activation and latency reversing agents induce HIV expression in only a fraction of infected cells [41–47].

We also observed a surprising degree of heterogeneity in expression of some human genes between single cells in the same clonal cell line. Some of this heterogeneity could reflect variable performance or imprecision in the assays, particularly for transcripts present at very low copies, although we observed fairly consistent results for RNA controls run outside of the C1 platform (HIV standards or extracted cellular RNA) and with repeat testing of aliquots from single cell cDNA across multiple Biomark (PCR) runs. The heterogeneity among clonal cells could also reflect stochastic variations in transcription or biological differences between single cells, such as differences in epigenetics, the phase of the cell cycle, the cellular microenvironment, or other factors. Stochastic variations in cells of the same type can have major implications for cell fate decisions [48]. As cell lines are non-quiescent, cell cycling also could contribute to differences in expression of particular transcripts and subsequent steady-state levels of protein [49]. Furthermore, RNA polymerase II and many other transcription factors can interact with extragenic enhancer sites to produce noncoding enhancer RNAs (eRNAs) that play a vital role in transcriptional regulation [50] and potentially cell-cycle progression [49]. Egr1, SP1, and p53 have been recently identified to contain eRNA motifs [49], which may contribute to differing levels of RNA expression at the single cell level. This single cell variation should be considered for future research using these HIV-infected cell lines.

Several lines of evidence suggest that single cell expression of the cellular genes we studied, including antiviral factors and genes implicated in latency, was more highly associated with genetic background than with level of HIV expression. First, cell lines derived from the same parental strain (ACH-2 and 8E5, unstimulated and activated J-Lat 9.2 cells) clustered together despite very different levels of HIV expression. Second, the cellular genes seemed to drive most of the differences in the principal component analysis. Third, there was a lack of consistency across cell lines in the human transcripts that correlated with HIV transcript levels. Although we only measured cellular gene expression in one of the 5 J-Lat clones for which we studied the bulk cell HIV transcription profile, the fact that all 5 J-Lat clones showed a very similar HIV transcription profile suggests that genetic background (or alternatively the virus, infection conditions, and method to select for infected cells) may have a strong influence on the mechanisms of latency. Other single cell transcriptomic studies have also shown that cellular genetic background drives much of the differential expression observed [51, 52]. Sophisticated bioinformatics tools are required to distinguish donor-independent, HIV-modulated changes to gene expression, which will be an inherent challenge for all such studies.

Our targeted single-cell approach enabled us to study cellular factors of interest, particularly those with reported associations with HIV infection and latency (Additional file 4: Table S2). We hypothesized that chronically-infected cell lines may exhibit altered expression of antiviral-associated genes, such as essential sensors of viral RNA (RIG-I and MDA5), to support the persistence of HIV. Although we did not observe a significant negative correlation between RIG-I and HIV transcripts, it is possible that lower abundance of RIG-I might contribute to impaired RNA-sensing capability in the setting of ongoing viral transcription (as occurs in 8E5 and ACH-2), permitting HIV persistence in the absence of robust PRR signaling. In addition to lower basal RIG-I expression, HIV protease reportedly sequesters RIG-I to evade the RIG-I signaling cascade [53], which could also contribute to impaired innate immune responses to HIV transcription. Interestingly, J-Lat 9.2 cells did not express IFNα, further suggesting that despite upregulation of RIG-I, the cellular response to infection could be drastically attenuated.

Reverse-transcribed HIV DNA can trigger C-GAS-mediated activation of STING, an adaptor protein that subsequently induces Type I interferons and cytokines [54]. Although we did not detect IFNα in J-Lat 9.2 irrespective of activation, the increase in STAT1 that correlated with polyadenylated HIV transcript expression (PolyA: P < 0.004; Additional file 12: Fig. S9) suggests that activation of the JAK/STAT pathway may be driven by other IFNs such as IFNγ (IFI16 is upregulated upon J-Lat 9.2 activation; Fig. 4a) [55] or other cytokines [56]. The low and variable expression of cellular antiviral defenses may have contributed to or selected for survival of these cell lines after infection, especially for the ACH-2 and 8E5 cells that express more HIV. It is possible that a similar phenomenon contributes to the survival of some HIV-infected cells in vivo, especially those that go on to form the latent reservoir. Several reports have described impairment in IFN-mediated antiviral responses in HIV-infected individuals, and PBMCs from HIV-positive individuals show impaired IFN-stimulated responses to transfected HIV [57–60].

In J-Lat 9.2 cells, we found that expression of many cellular genes, including housekeeping genes, correlated with multiple HIV targets, suggesting a general association between overall levels of cell transcription and viral transcription, which makes sense given the mechanism of transcriptional interference in this cell line. However, a subset of the genes showed higher r values and may be more closely associated with HIV transcription, including CCR5, BCL11B, CDK9, CREBBP, and STAT1 (*P *< 0.00012 for all; Fig. 5, Additional file 12: Fig. S9). Some of these correlations are expected. CDK9 together with cyclin T1 comprise P-TEFb, which associates with HIV TAR [61] and is a target for HIV Tat-mediated transactivation [62]. Similarly, it is understood that CREBBP is recruited by HIV Tat to the 5^′^-LTR to enhance transcription [63]. However, somewhat paradoxically, the expression of BCL11B, which represses transcription from the LTR to mediate HIV-silencing [64], was also associated with TAR and Gag expression in J-Lat 9.2 cells (*P *< 4.6 × 10^−5^; Fig. 5, Additional file 12: Fig. S9). Interestingly, we also observed a correlation between STAT1 RNA expression and TAR and PolyA in J-Lat 9.2 (*P *< 0.004; untreated and activated, respectively), suggesting the induction of the Type 1 IFN JAK/STAT pathway in these chronically-infected cells, in line with data from other J-Lat clones implicating the JAK/STAT pathway in latent virus reactivation [65].

Despite the correlations in J-Lat 9.2 cells, we did not observe a consistent relationship between expression of any cellular gene in our panel and HIV transcripts across all cell lines. For instance, CD69 correlated positively with HIV expression in J-Lat 9.2 and activated J-Lat 9.2, but not in productively-infected 8E5, or in ACH-2, where there was a trend toward a negative correlation (Fig. 5a). Our finding that no cellular gene consistently correlated with HIV expression was not unexpected, given that these cell lines exhibit fundamental differences in their mechanisms of latency and cellular gene expression.

Chronic infection alters expression of many cellular genes relative to the parental cell line, such as genes encoding transcription factors, proteasome components, factors that control immune function, and apoptosis in ACH-2 relative to its parental cell line, A3.01 [66]. The upregulation of anti-apoptotic genes including chaperonins, heat shock proteins, histone deacetylases and proteasome subunits has been implicated in contributing to the maintenance of HIV latency in ACH-2 [67]. Activation of the J-Lat 9.2 cells also led to upregulation of many genes, including those involved in pathogen sensing, immune responses, and anti-apoptotic functions, suggesting that activation and/or viral expression triggers a number of signaling cascades. Within the ACH-cells, 8E5 cells, and activated J-Lats, expression of multiply-spliced Tat-Rev was associated with differential expression of cellular genes involved in apoptosis and survival. Cellular and immune defenses can trigger apoptosis in response to viral products, while anti-apoptotic pathways may contribute to the survival of immortalized cell lines, especially in the face of productive HIV infection. Emerging evidence suggests that anti-apoptotic pathways may also contribute to the survival of HIV-infected cells and persistence of proviral clones in vivo [68–70], and these pathways may be targets for new therapies aimed at killing infected cells and curing HIV [71].

The limitations of this study should be acknowledged. Subculturing of cell lines has been reported to result in perturbed morphology, growth rates, protein expression and response to stimuli [72–74]. Despite the low passage of cells used in this study [2–5], it is possible that aberrant changes occur on a shorter scale in cell lines, and ongoing replication and integration into unique sites [28] could contribute to the variability in gene expression that we observe at the single cell level. These data underscore the need for minimizing subculturing of cell lines prior to undertaking experiments.

As measured by HIV DNA levels per million cells, we observed less than one HIV provirus per cell in some of the cell lines (including 8E5 and J-Lat 6.3, 8.4, and 15.4). It is possible that some cells have lost the HIV provirus or harbor mutated proviruses, consistent with previous reports [28, 31, 32]. HIV DNA levels were consistent across 5 different regions (aside from the expected twofold excess of targets confined to the LTR, which is present at each end of the provirus), arguing against selective mutation or assay insensitivity in one proviral region. Moreover, frequencies of < 1 provirus/cell were observed with two widely-accepted methods of normalizing to cell numbers. However, the cell equivalents as determined by DNA mass and TERT tended to exceed the pre-extraction cell counts (DNA mass was closer), which suggests error in the NanoDrop or TERT measurements. It is possible that some cell lines have a duplication of the TERT gene and/or some chromosomes, especially if the cells are polyploid or a substantial proportion of cells are progressing through the cell cycle. Duplication of the TERT gene and/or chromosomes that do not contain the provirus could lead to underestimation of the HIV DNA (infection frequency) as normalized to TERT or DNA mass. In support of the latter explanation, the infection frequency of the 8E5 cells was higher when measured in single cells (43/44) than bulk cells. However, the sensitivity to detect an infected cell may be higher in the single cell assays due to the inclusion of 7 different HIV targets, the ability to detect RNA as well as DNA, and the inclusion of a pre-amplification step prior to PCR.

A limitation inherent to all transcriptomic-based studies is the reality that protein expression is influenced by many post-transcriptional processes, including RNA processing (alternative/differential splicing); RNA stability; translational regulation (presence of regulatory elements in the 5′ untranslated region or tRNA availability [75]); and differential stability, regulation, and post-translational modification of protein species [49]. RNA expression does not infer levels of protein generated, nor does it provide insight into the phosphorylation states of their residues [49] or functionality, which can limit our interpretation of some observations. For instance, although we observed a correlation between STAT1 RNA expression and HIV transcripts in J-Lat 9.2, HIV proteins can inhibit the phosphorylation of STAT1 protein [76] or target STAT1 for proteosomal degradation [77] to perturb antiviral responses via the JAK/STAT pathway. As another example, 8E5 cells have been shown to transcribe CD4 RNA at levels similar to their parental CD4+ cell line, but show low surface expression of CD4 because the protein is sequestered with HIV Env in the rough endoplasmic reticulum [78]. Similarly, although J-Lat 9.2 exhibited relatively high CD3 RNA levels (Fig. 4a) in both non-activated and activated states, it is possible that the subsequent protein expressed may be limited or less functional. J-Lat clones 6.3, 8.4 and 11.1 are unresponsive to activation using αCD3/αCD28 despite strong responses to phytohemagglutinin, which also stimulates signaling cascades involving TCR/LCK/p38 activation [20]. This finding in closely related J-Lat clones could point to suboptimal function of CD3 within these clones.

RNA and protein levels can also be influenced by other factors mentioned above, including intrinsic ‘transcriptional noise,’ epigenetics, cell cycle, and the microenvironments of cells [79], which were not assessed in this study. Despite these limitations, our data provide valuable insights into the relationship between the HIV transcription profile and transcriptome of widely-used cell lines.

## Conclusions

Although primary cell models are increasingly employed in studies of HIV latency, continued research efforts utilize HIV-infected cell lines to evaluate new assays for HIV persistence, investigate the molecular mechanisms that underlie the establishment/maintenance of HIV latency, and test new therapies designed to disrupt latency [80–87]. In order to best employ these cell lines, it is critical to understand how they differ from each other and from cells from ART-treated individuals in the mechanisms underlying latent HIV infection. We used novel HIV transcription profiling and leading-edge single cell technology to compare widely-used cell line models of latent and productive HIV infection. We found marked differences between each cell line and cells from ART-treated individuals in the HIV transcriptional blocks underlying latency, and between cell lines in single cell expression of viral and cellular genes. Furthermore, despite the clonality of the cells, viral genetics, and integration sites, single cell-data revealed significant variability in viral and cellular expression profiles. Use of these cell lines for HIV research to address a particular research question should take into account the differences in viral and cellular gene expression patterns observed here. Despite the differences between HIV-infected cell lines, some findings were shared across cell lines, such as reduced expression of cellular antiviral factors and a relationship between expression of multiply-spliced HIV RNA and expression of human genes involved in apoptosis or survival. These factors may contribute to the maintenance and persistence of the latent reservoir in vivo. To further investigate the mechanisms of latency and develop new therapies that more specifically and more efficiently disrupt latent HIV infection, novel single cell methodologies such as those described in this study should be applied to primary cell latency models and unstimulated cells from ART-treated individuals.

## Methods

### Study participants

The study participants were HIV-infected adults on suppressive ART from two cohorts (median age = 51; median CD4 count = 611 cells/mm^3^; median years of suppression = 5). Cryopreserved PBMCs (10^7^ cells) were available from 9 study participants in the Reservoirs and Drug Levels (RADL) study [10]. CD4+ T cell pellets, which had been isolated using negative selection from the blood and either frozen immediately or after activation for 2 days with anti-CD3/CD28 and antiretrovirals, were available from 7 ART-suppressed study participants recruited prospectively and sequentially from the San Francisco VA Medical Center.

### Cell culture and activation

Cell lines were obtained through the NIH AIDS Research Reagent Program (NIH ARRRP), Division of AIDS, NIAID, NIH: ACH-2, 8E5 and U1 (U937) from Dr. Thomas Folks, and J-Lat Full Length Clones (6.3, 8.4, 9.2 and 15.4) from Dr. Eric Verdin. J-Lat Full Length clone 5A8 was obtained through Dr. Warner Greene. J-Lat full length 5A8, 6.3, 8.4, 9.2 and 15.4 cells were cultured in Roswell Park Memorial Institute (RPMI) 1640 medium (Invitrogen Life Technologies, Carlsbad, CA, USA) supplemented with 10% fetal bovine serum (FBS, Sigma-Aldrich, St. Louis, MO), 100 U/ml penicillin, 100 mg/ml streptomycin and 2 mM l-glutamine (Invitrogen, Carlsbad, CA). ACH-2 cells were cultured in RPMI 1640 supplemented with 10% FBS, 10 mM HEPES, and 2 mM l-glutamine. HIV-1 infected U937 cells (U1) were cultured in RPMI 1640 containing 10% FBS and 2 mM l-glutamine. HIV-1 LAV infected 8E5 cells were cultured in RPMI 1640 supplemented with 10% FBS. Cells were incubated at 37 °C, 5% CO_2_, and passaged twice a week following NIH ARRRP recommendations. J-Lat 5A8 and 9.2 cells were activated in bulk by culture for 24 h with 10 ng/ml phorbol 12-myristate 13-acetate (PMA) and 1uM Ionomycin (Sigma). Cells were cultured in parallel with unstimulated J-Lat (clone 9.2 and 5A8) cells of the same passage. Following stimulation, J-Lat 9.2 and 5A8 cells (unstimulated and stimulated) were prepared for bulk HIV RNA and DNA analysis, and J-Lat 9.2 cells (unstimulated and stimulated) loaded into the either 5–10 µm C1 integrated fluidic circuit (IFC) [unstimulated cells] or 10–17 µm C1 IFC [stimulated cells] as described.

### Bulk HIV RNA and DNA quantification

For all the cell lines, at the lowest passage number as possible (< 5 in all cases), when the viability was above 90% and after 24 h of activation using PMA/Ionomycin, aliquots of 2 × 10^6^ cells were immediately frozen as cell pellets. Total cellular RNA and DNA were isolated using Trireagent following manufacturer’s instructions. RNA and DNA concentrations were quantified using UV spectrophotometry (NanoDrop 1000). TAR levels were quantified by 3-step polyadenylation-RT-ddPCR [9, 88], while Read-through, LongLTR, Pol, Nef, PolyA and Tat-Rev transcripts were quantified by 2-step-RT-ddPCR, as previously described [9, 10], except for ACH-2, for which the TAR sequence was amplified using a different probe (5′ AGCCTGGGAGTTC 3′) to avoid sequence mismatch caused by the mutation at nucleotide + 37 (C⟶T) in the TAR loop of ACH-2 cells [18]. Cellular DNA was fragmented using QIAshredder columns and used to quantify total HIV DNA for TAR, Read-through, LongLTR, Pol and Nef, using the same primer–probe sets used for HIV RNA transcripts, and the human gene TERT. All samples were tested in duplicate. “No RT” reactions and PBMCs from HIV negative donors were tested in parallel to check DNA contamination or as negative controls, respectively. HIV RNA transcripts and HIV DNA were normalized to million cells using DNA mass (by NanoDrop, assuming 1 μg DNA equals 160,000 cells) and the TERT gene copies in the total extracted DNA, the resuspension volumes of the DNA or RNA, and the proportions going into the RT and/or ddPCR reactions. The average levels of each HIV transcript per provirus were calculated by dividing levels of each HIV RNA by levels of the LongLTR DNA (present in one copy per provirus), and by dividing each HIV RNA by levels of the same HIV DNA region (corrected for 2 LTR regions per provirus).

### Primer selection

Assay targets were selected based on their reported association with T cell function, HIV infection, and HIV latency, and included genes for housekeeping transcripts, T cell function and phenotype, HIV coreceptors, markers of activation/proliferation/senescence, cytokines, genes involved in apoptosis or pyroptosis, antiviral or restriction factors, and genes implicated in HIV latency, transcription, mRNA end processing, and splicing. Genes were selected from the catalogue of inventoried and custom TaqMan™ primers and probes for qPCR (Invitrogen Life Technologies). In instances where multiple TaqMan™ primer/probes sets were available for the same target, the following selection criteria were applied: (1) primers spanned exon boundaries to selectively amplify mRNA, (2) size of amplicon (120 bp or less), and (3) highest number of reference sequences available. In some cases, not all of these criteria could be met, in which case the ‘preferred’ option, as recommended by Life Technologies was selected.

### Custom HIV primers

Custom TaqMan™ primer/probe sets were designed based on transcription profiling assays [9, 89]. Primer and probe sequences were the same as those described previously for ddPCR and RT-ddPCR [9, 90]. The performance of these assays in the single cell C1 platform was assessed using two standards: (1) an unspliced genomic HIV RNA (virion) standard, which detects all assays except for multiply-spliced Tat-Rev; and (2) an in vitro transcribed, synthetic, read-through, multiply-spliced, polyadenylated HIV RNA standard, which detects all assays except Gag and Pol.

All primer/probe sets were assessed based on efficient amplification (where the signal doubles with each PCR cycle), linearity (where the final signal is proportional to input RNA) and performance using bulk donor PBMCs and RNA (~ 200 cells). Negative and No-RT controls were routinely included in each experiment. Negative controls were consistently negative for amplification.

### Single cell partitioning, RT, and preamplification using the C1 assay system

Cells were counted using an automated counter (TC-20, Bio-Rad, Hercules, CA) and manual counting using a hemocytometer. Cells were washed twice in PBS and resuspended at a concentration of 1200 cells/µL. Cells (6 µL) and reagents (Ambion Single cell-to-CT qRT-PCR kit, Thermo Fisher, Waltham CA) were loaded into the C1 integrated fluidic circuits (IFCs) as per manufacturer’s recommendations.

Validations were performed to determine optimal cell concentration, cell buoyancy, and input for highest single-cell capture rate. Cell capture was evaluated by microscopy directly following ‘Cell Load’ script. Single, healthy-appearing cells were selected for downstream analysis post-C1 protocol. Cell lysis, reverse transcription and cDNA pre-amplification were carried out using the C1 instrument (Fluidigm Corp., San Francisco, CA).

The IFC size selected was based on expected cell size: 5-10 µm chips were used for unstimulated J-Lat (clone 9.2), 8E5, U1 and ACH-2, whereas 10-17 µm IFC were selected for stimulated J-Lat cells. Following C1 assay completion, cDNA was harvested and diluted 1 in 8 in C1 DNA Dilution buffer (Fluidigm Corp.) and stored at − 20 °C prior to use.

### Gene expression analysis using Biomark HD system

TaqMan™ primers/probes (Thermo Fisher) and sample cDNA were dispensed into discrete inlets of a 96 × 96 Dynamic Array microfluidic chip (Fluidigm Corp.) as per manufacturer recommendations.

Forty cycles of PCR were performed using the Biomark instrument (Fluidigm Corp). Data were analyzed using the Fluidigm Real-time PCR Analysis software suite (version 4.1.3, Fluidigm Corp.) and the SINGuLAR Analysis Toolset Software (version 3.6.2, Fluidigm Corp.) in R (version 3.0.2, open source) to validate assay performance and in instances where ‘failed’ reactions were reported, manual verification was performed. Where fluorescence data displayed characteristics of logarithmic amplification, and the expected “Ct” (threshold cycle) was achieved, reactions were designated “passed”.

### Statistical analysis

Single cell analyses were performed using R. Principal component analysis was performed using the FactoMineR v1.41 package [91], and t-SNE analysis was performed using the Rtsne v0.15 package [92]. Differential gene expression analysis was performed using Poisson generalized linear model as implemented in Seurat v2.3.4 [93, 94]. All correlation analyses were performed using Spearman correlations. P-values were adjusted for false discovery rate (FDR) [95]. Hierarchical clustering analyses were performed using Ward’s method [96] as implemented in pheatmap v1.0.12. All plots were generated using the ggplot2 v3.1 and pheatmap v1.0.

## Supplementary information

**Additional file 1: Fig. S1.** Bulk cell HIV DNA and RNA levels. **A** Total HIV DNA copies per million cells (normalized by DNA mass). **B** Total HIV DNA copies per million cells (normalized by TERT). **C** HIV RNA levels normalized to million cells (by DNA mass). **D** Cell-associated HIV RNA copies per provirus, as normalized by ratio of each HIV RNA to the corresponding HIV DNA region. For PBMCs, CD4+ T cells, and activated CD4+ T cells from HIV-infected ART-suppressed individuals (B, C), each individual is shown as a dot, the column height indicates the median, and bars represent 25–75%.

**Additional file 2: Fig. S2.** Progression through HIV transcription stages. This schematic shows relative levels of HIV transcription initiation, elongation, completion and multiple splicing quantified in the myeloid cell line U1; the lymphoid cell lines ACH2, 8E5, J-Lat clones and activated J-Lat clones; and in PBMCs, CD4+ T cell and activated CD4+ T cells from HIV-infected ART-suppressed individuals. The scale depicts the maximal block to transcription (red) to no transcriptional block (green). For each cell line, the blue arrow indicates the comparative progression through/block to transcription at each stage.

**Additional file 3: Table S1.** caHIV RNA transcript ratios.

**Additional file 4: Table S2.** Complete assay panel.

**Additional file 5: Fig. S3.** Sensitivity for HIV RNA in the single-cell Biomark HD platform. Each row represents a single sample. For donor PBMCs, the equivalent of 10 cells (RNA) was added to each reaction as a negative control. Two standards were used to assess the sensitivity of each HIV assay: a synthetic multiply-spliced HIV RNA standard (which contains TAR, LongLTR, Nef, PolyA, and Tat-Rev but not Gag or Pol) and an HIV virion RNA standard (which contains TAR, LongLTR, Gag, Pol, Nef, and PolyA, but much lower levels of Tat-Rev). Both standards were added to each independent Biomark assay at 5, 10, 50 and 500 copies. All assays except PolyA could be detected down to 5 copies, but PolyA was less efficient than the other HIV assays in this platform.

**Additional file 6: Fig. S4.** Reproducibility of independent Biomark HD experiments. **A** Aliquots of cDNA from individual cells were tested in separate Biomark HD experiments. Y and X axes show the expression levels (40-C_T_) of each HIV target (left plot) and cellular gene (right plot) from separate Biomark HD runs. R values are from Spearman correlations. **B** Dropout occurrence for HIV and gene expression assays. The table shows all cases for which an HIV or cellular target was detected in one Biomark HD experiment but not another, along with the particular cell line and expression levels.

**Additional file 7: Table S3.** Number of single-cells analyzed across cell lines.

**Additional file 8: Fig. S5.** T-distributed stochastic neighbor embedding (tSNE) plot. tSNE plot of gene expression profiles representing the clustering of individual cells post-ComBat adjustment. ComBat adjustment was performed to control for batch effects. Single-cells for each cell line are indicated by color and symbols denote independent assays (batch).

**Additional file 9: Fig. S6.** Principal component analysis. Correlation coefficients of top principal components and 95 genes. Each row represents a different dimension in the PCA analysis; each column indicates a different cellular gene or HIV target. No expression of TIGIT was detected and was subsequently excluded from further analysis. The color scale (right) denotes Pearson coefficients. Dendrograms (above) show unsupervised clustering.

**Additional file 10: Fig. S7.** Single cell variation in cellular and HIV expression. Cells are grouped on basis of cell line. Each vertical line represents a single cell. All cellular (89) and HIV (7) targets are shown on separate rows. The blue to red scale (right) denotes expression levels (40-C_T_). Dendrograms (left) show unsupervised clustering. Each cell line and category of gene target (antiviral/restriction factor, HIV transcription/latency, T cell phenotype/function, housekeeping, HIV target) is indicated by a different color.

**Additional file 11: Fig. S8.** False correlations driven by non-detection of cellular and HIV targets. Shown are correlations between Tat-Rev and expression of C-GAS (left panel) and HLA-DR (right panel) in U1 cells.

**Additional file 12: Fig. S9.** Positive correlations between expression of cellular and HIV targets in J-Lat 9.2 (untreated and activated). Positive correlations between cellular and HIV targets in **A** non-activated J-Lat 9.2 and **B** activated J-Lat 9.2. R and p values are from Spearman correlations.

**Additional file 13: Fig. S10.** Differentially expressed genes in unstimulated vs. activated J-Lat 9.2 cells. Each dot represents a separate gene or HIV target. The X axis represents the log_2_ fold change and the y-axis denotes the − log_10_(P value).

## Data Availability

The published article and its supplementary files show all relevant analyzed data. The raw data used during the current study are available from the corresponding author on reasonable request.
