# Supplementary figures and images for: Heterogeneity in HIV and cellular transcription profiles in cell line models of latent and productive infection: implications for HIV latency
Source: Retrovirology. 2019 Nov 11;16:32. doi: 10.1186/s12977-019-0494-x (PMC6849327; doi:10.1186/s12977-019-0494-x)

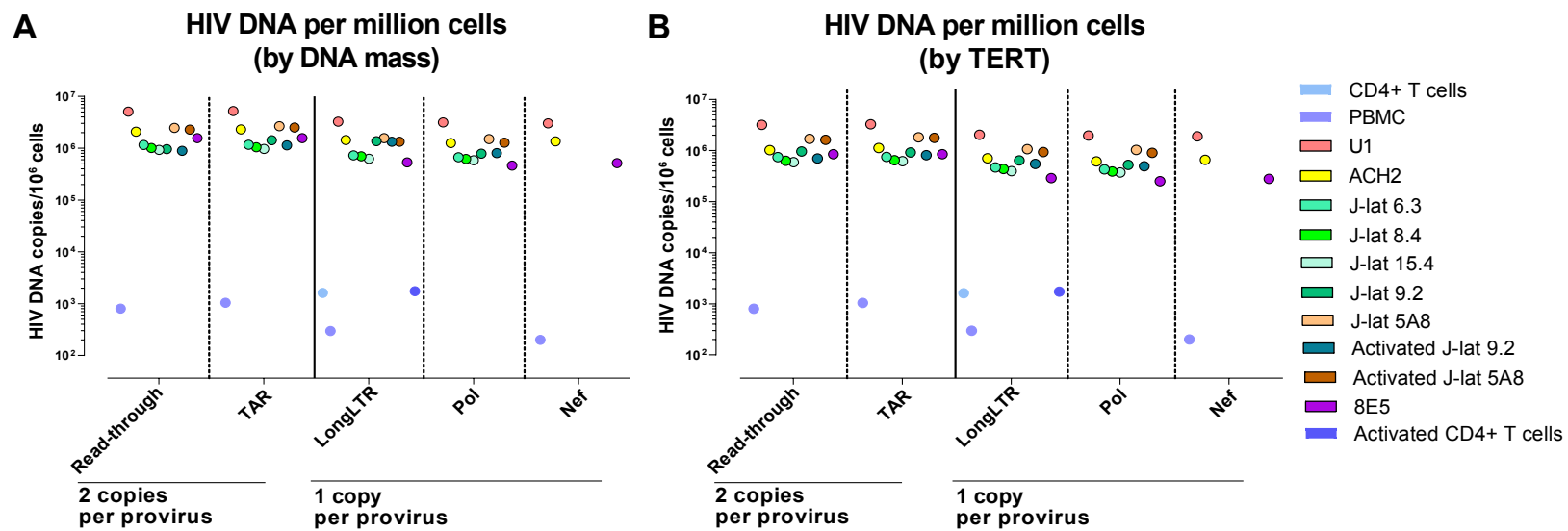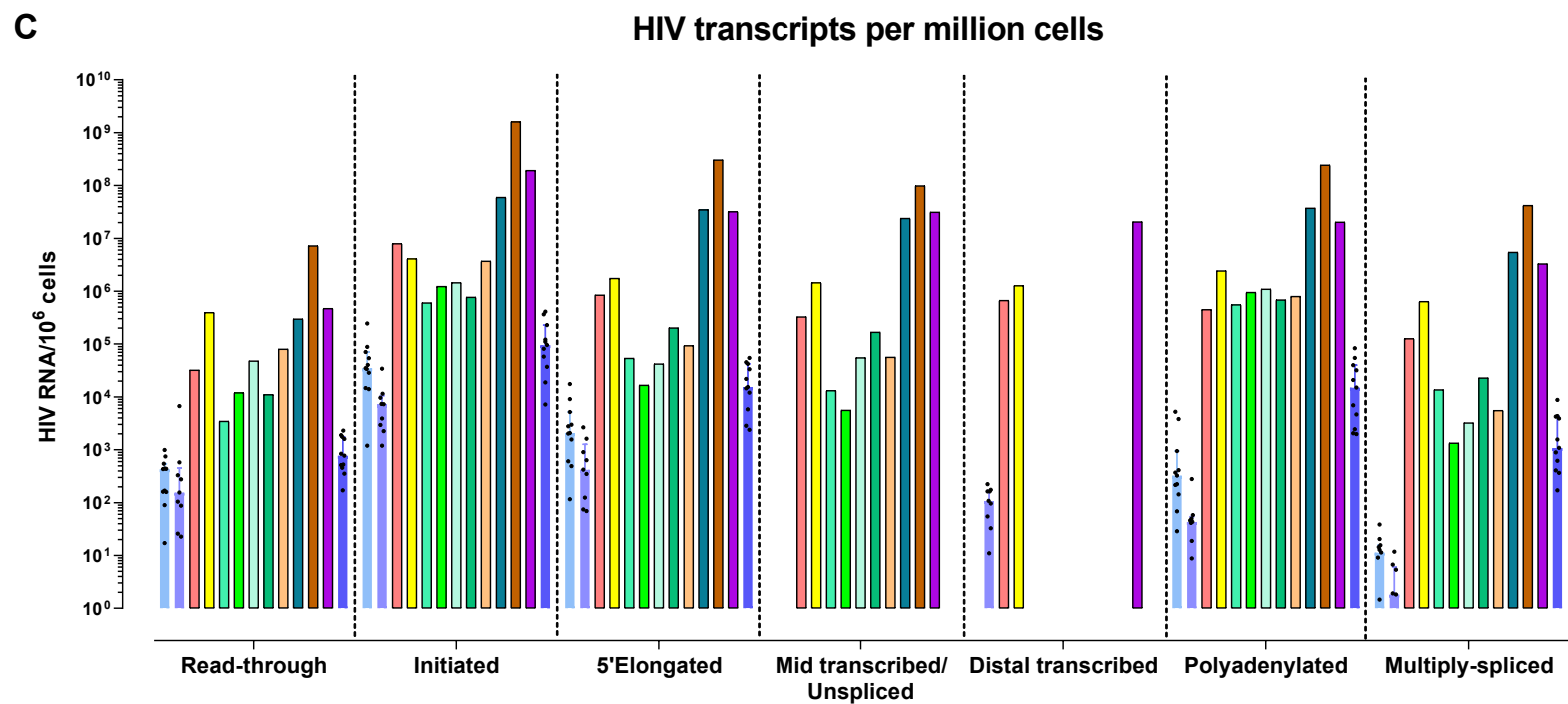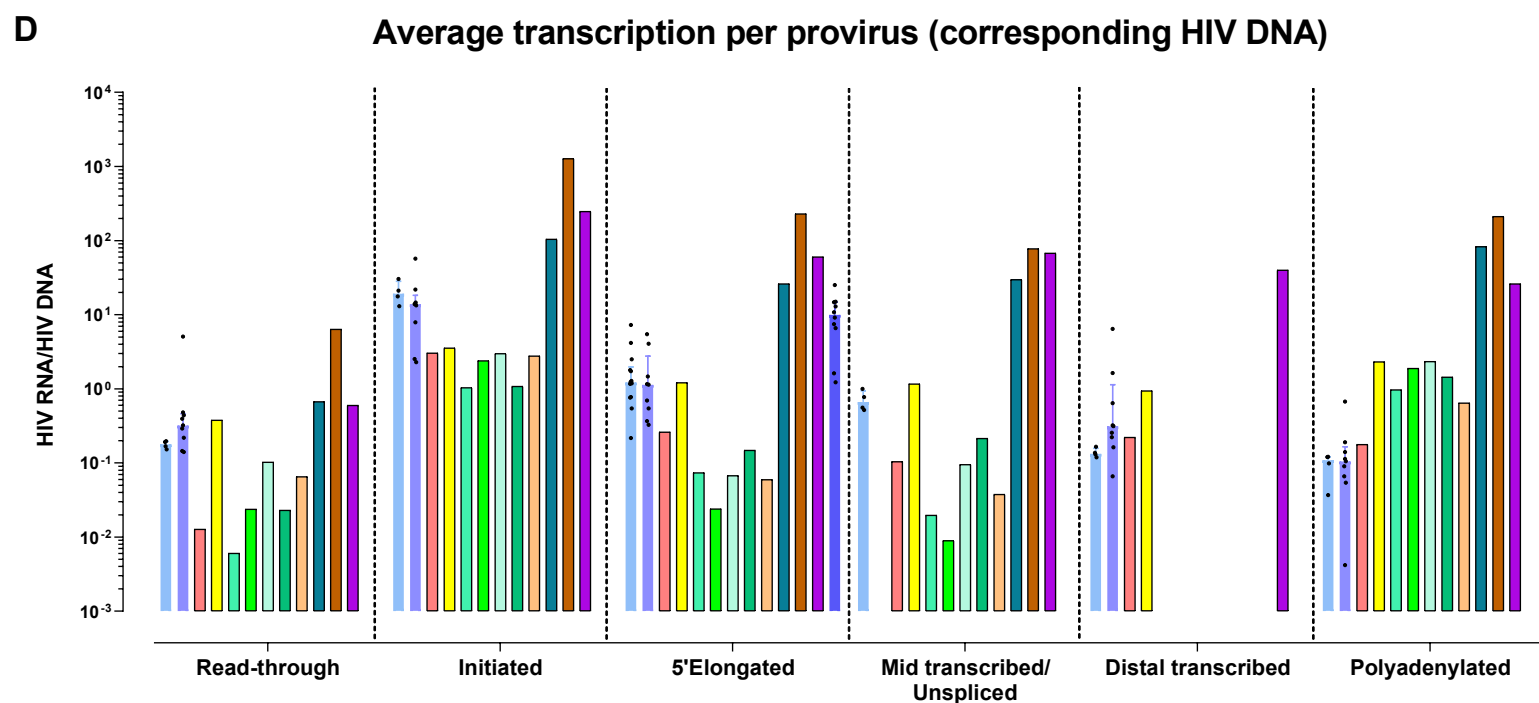

Supplement: Supplementary file 1 — Additional file 1: Fig. S1. Bulk cell HIV DNA and RNA levels. A Total HIV DNA copies per million cells (normalized by DNA mass). B Total HIV DNA copies per million cells (normalized by TERT). C HIV RNA levels normalized to million cells (by DNA mass). D Cell-associated HIV RNA copies per provirus, as normalized by ratio of each HIV RNA to the corresponding HIV DNA region. For PBMCs, CD4+ T cells, and activated CD4+ T cells from HIV-infected ART-suppressed individuals (B, C), each individual is shown as a dot, the column height indicates the median, and bars represent 25–75%. [file 12977_2019_494_MOESM1_ESM.pdf]

# Progression through HIV transcription stages

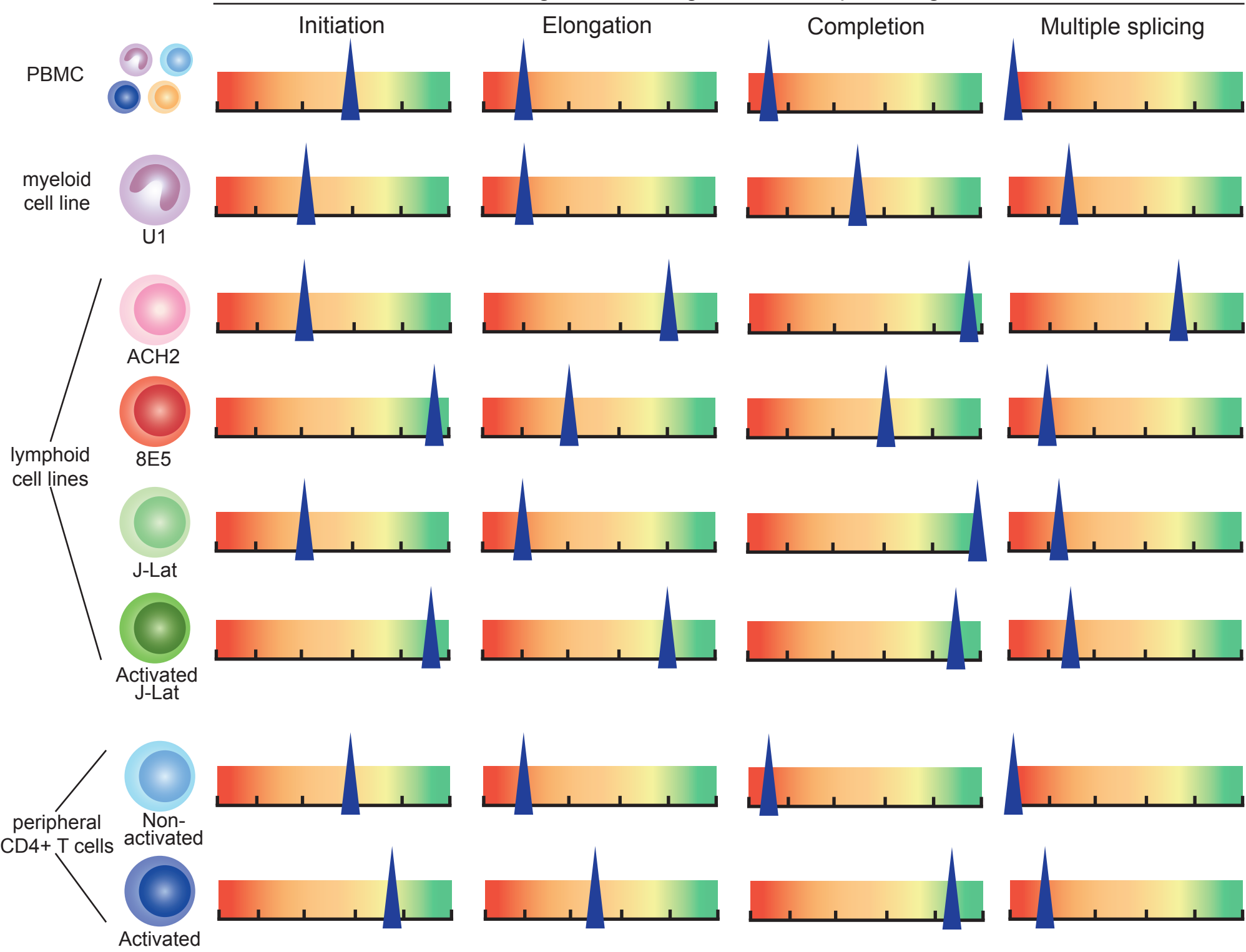

Supplement: Supplementary file 2 — Additional file 2: Fig. S2. Progression through HIV transcription stages. This schematic shows relative levels of HIV transcription initiation, elongation, completion and multiple splicing quantified in the myeloid cell line U1; the lymphoid cell lines ACH2, 8E5, J-Lat clones and activated J-Lat clones; and in PBMCs, CD4+ T cell and activated CD4+ T cells from HIV-infected ART-suppressed individuals. The scale depicts the maximal block to transcription (red) to no transcriptional block (green). For each cell line, the blue arrow indicates the comparative progression through/block to transcription at each stage. [file 12977_2019_494_MOESM2_ESM.pdf]

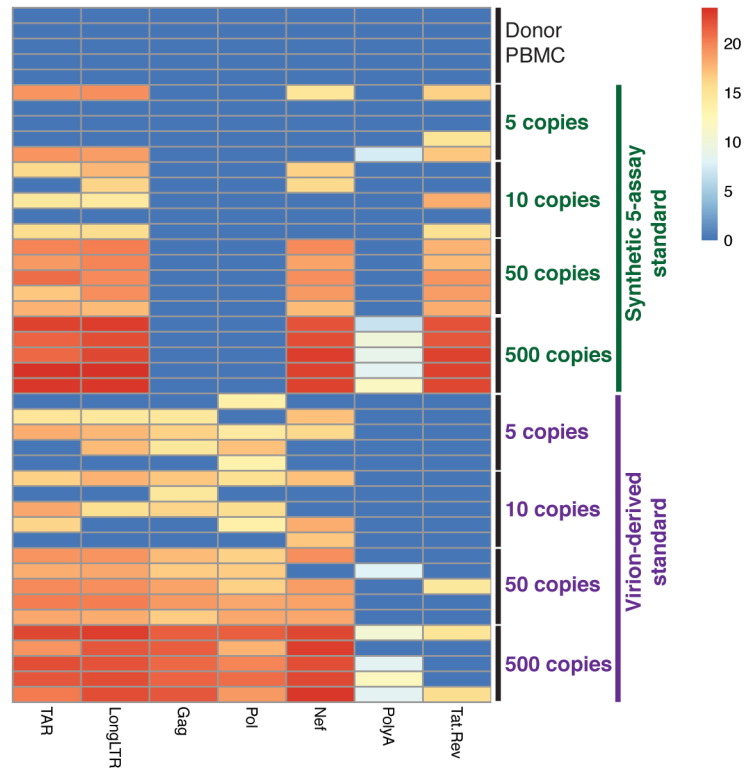

Supplement: Supplementary file 5 — Additional file 5: Fig. S3. Sensitivity for HIV RNA in the single-cell Biomark HD platform. Each row represents a single sample. For donor PBMCs, the equivalent of 10 cells (RNA) was added to each reaction as a negative control. Two standards were used to assess the sensitivity of each HIV assay: a synthetic multiply-spliced HIV RNA standard (which contains TAR, LongLTR, Nef, PolyA, and Tat-Rev but not Gag or Pol) and an HIV virion RNA standard (which contains TAR, LongLTR, Gag, Pol, Nef, and PolyA, but much lower levels of Tat-Rev). Both standards were added to each independent Biomark assay at 5, 10, 50 and 500 copies. All assays except PolyA could be detected down to 5 copies, but PolyA was less efficient than the other HIV assays in this platform. [file 12977_2019_494_MOESM5_ESM.pdf]

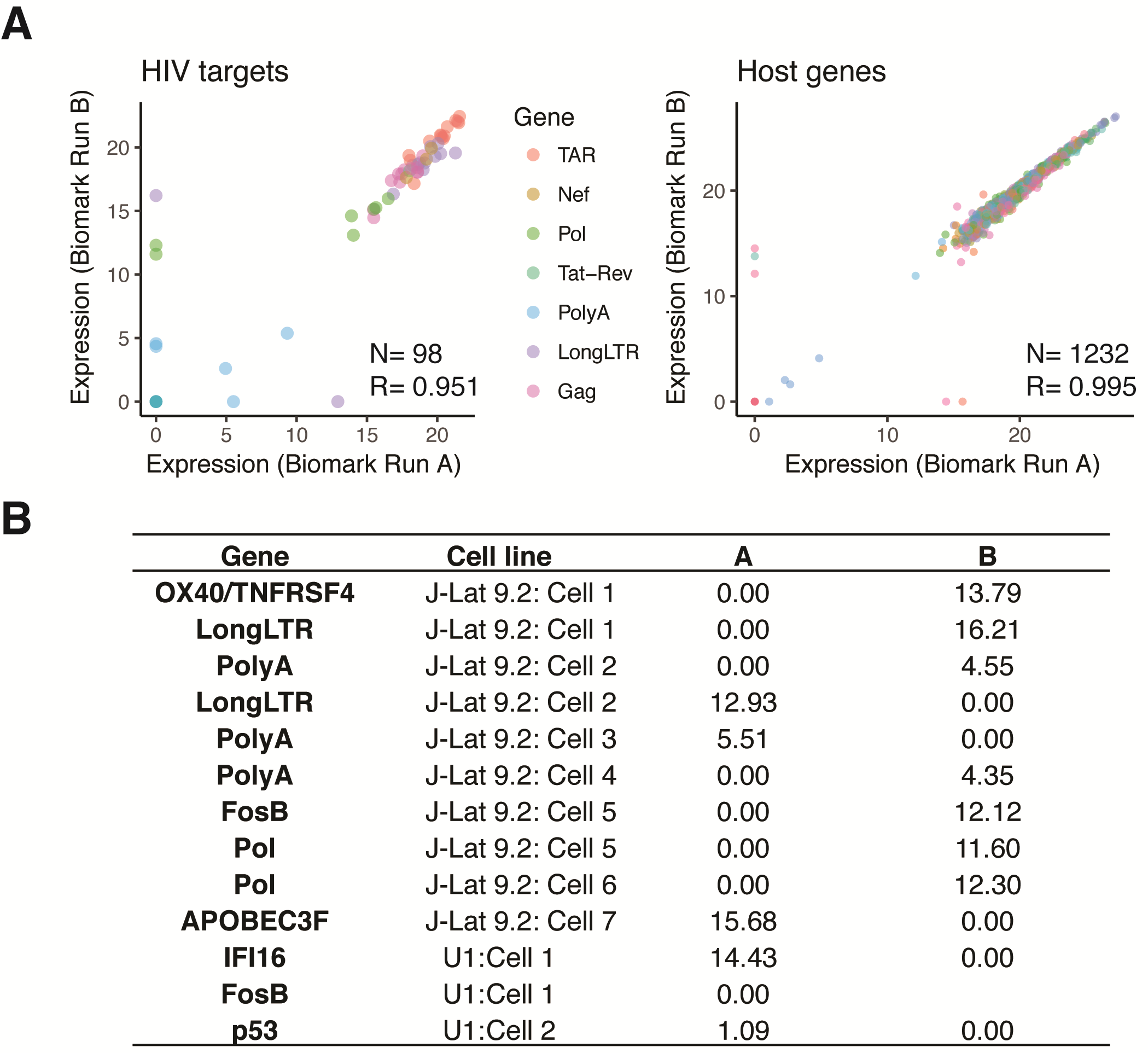

Supplement: Supplementary file 6 — Additional file 6: Fig. S4. Reproducibility of independent Biomark HD experiments. A Aliquots of cDNA from individual cells were tested in separate Biomark HD experiments. Y and X axes show the expression levels (40-CT) of each HIV target (left plot) and cellular gene (right plot) from separate Biomark HD runs. R values are from Spearman correlations. B Dropout occurrence for HIV and gene expression assays. The table shows all cases for which an HIV or cellular target was detected in one Biomark HD experiment but not another, along with the particular cell line and expression levels. [file 12977_2019_494_MOESM6_ESM.tif]

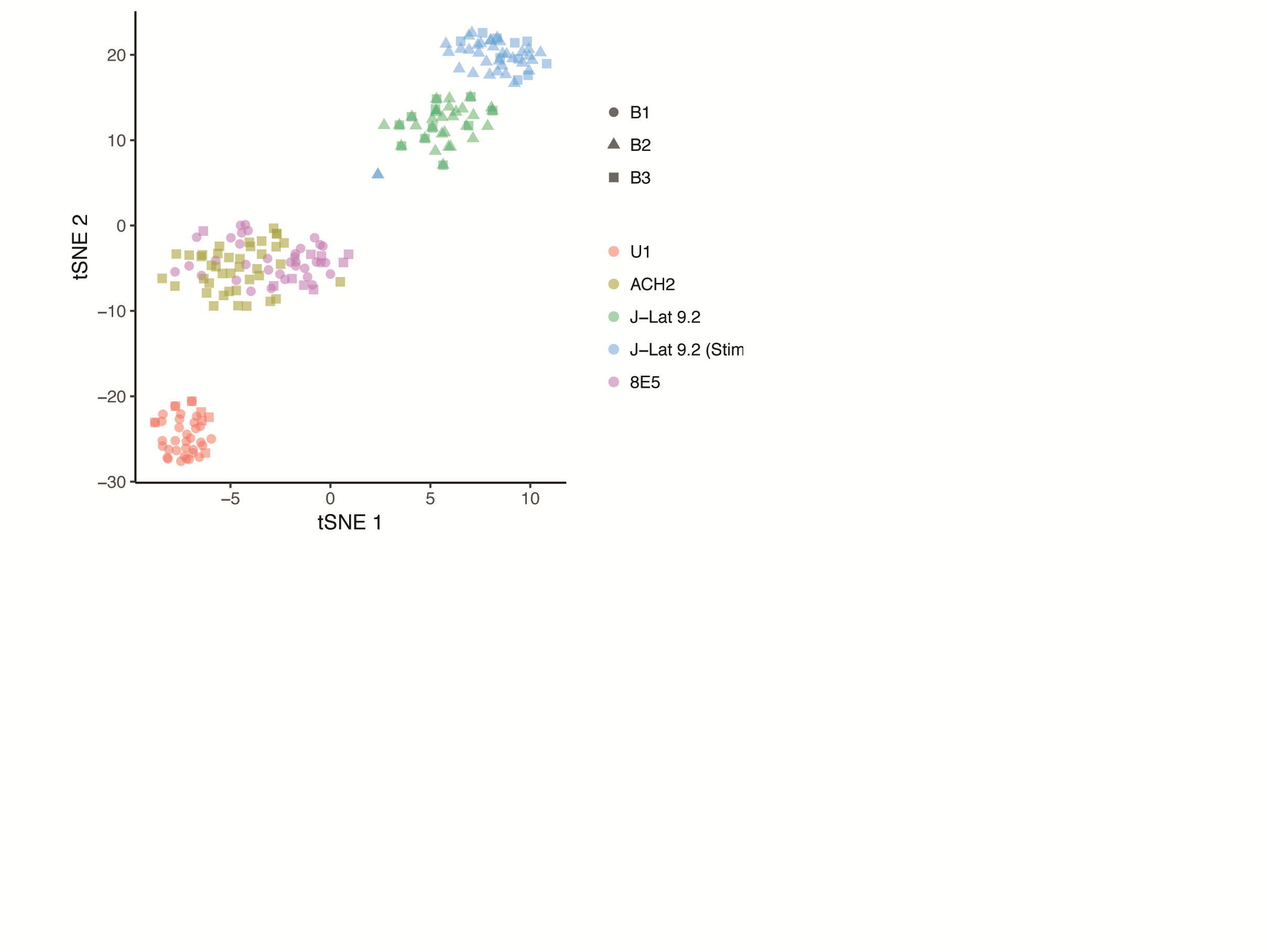

Supplement: Supplementary file 8 — Additional file 8: Fig. S5. T-distributed stochastic neighbor embedding (tSNE) plot. tSNE plot of gene expression profiles representing the clustering of individual cells post-ComBat adjustment. ComBat adjustment was performed to control for batch effects. Single-cells for each cell line are indicated by color and symbols denote independent assays (batch). [file 12977_2019_494_MOESM8_ESM.tif]

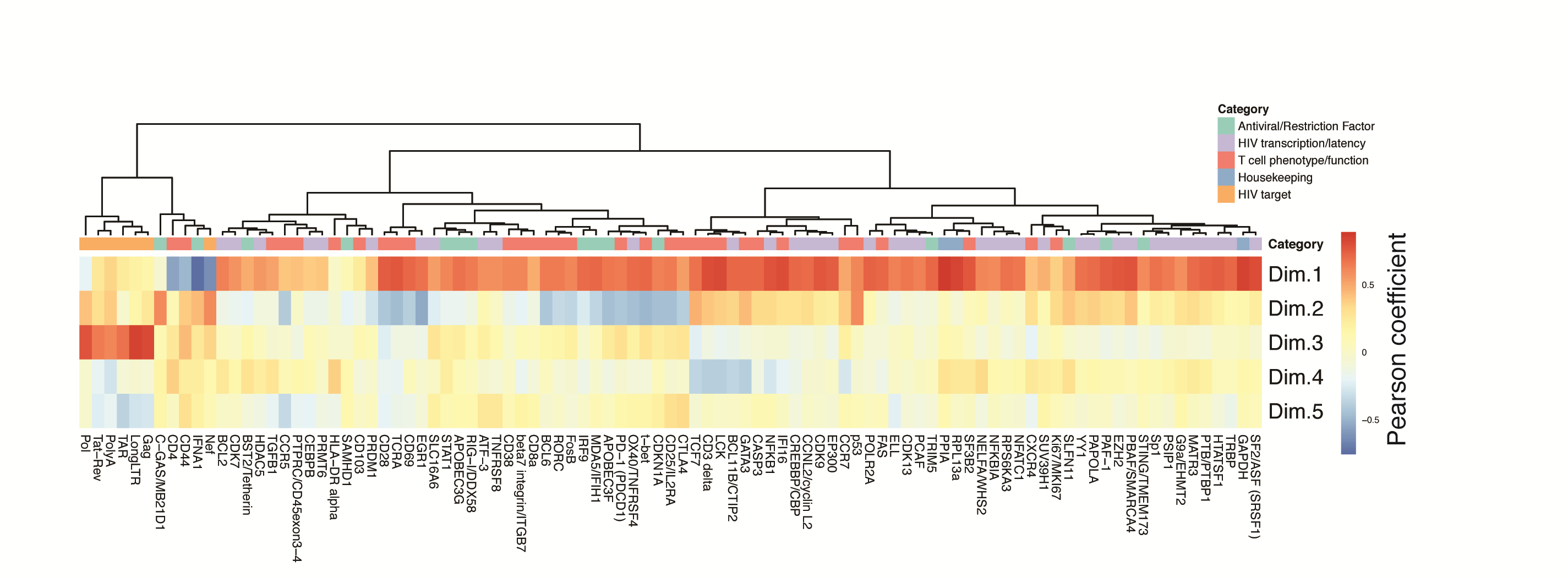

Supplement: Supplementary file 9 — Additional file 9: Fig. S6. Principal component analysis. Correlation coefficients of top principal components and 95 genes. Each row represents a different dimension in the PCA analysis; each column indicates a different cellular gene or HIV target. No expression of TIGIT was detected and was subsequently excluded from further analysis. The color scale (right) denotes Pearson coefficients. Dendrograms (above) show unsupervised clustering. [file 12977_2019_494_MOESM9_ESM.tif]

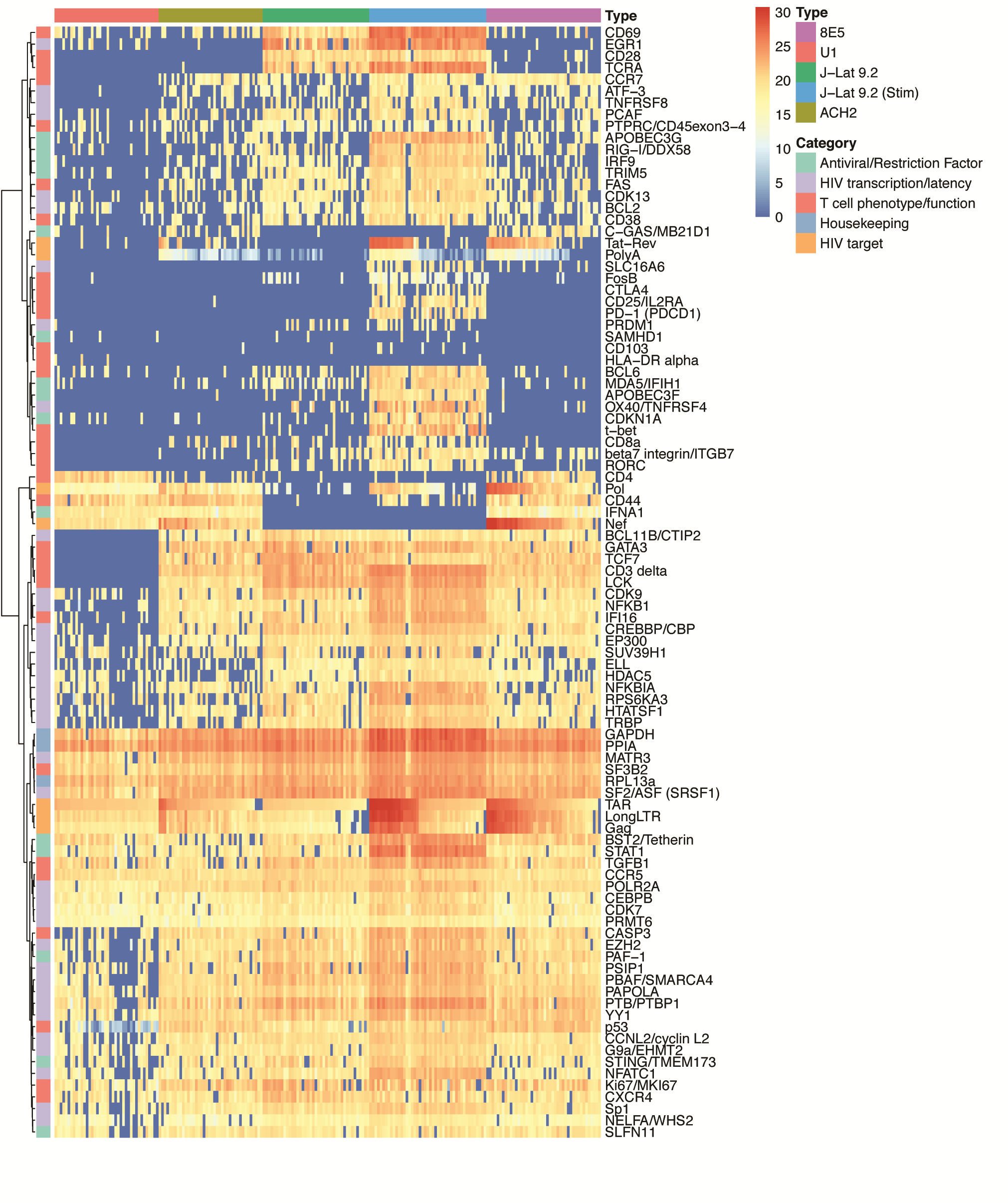

Supplement: Supplementary file 10 — Additional file 10: Fig. S7. Single cell variation in cellular and HIV expression. Cells are grouped on basis of cell line. Each vertical line represents a single cell. All cellular (89) and HIV (7) targets are shown on separate rows. The blue to red scale (right) denotes expression levels (40-CT). Dendrograms (left) show unsupervised clustering. Each cell line and category of gene target (antiviral/restriction factor, HIV transcription/latency, T cell phenotype/function, housekeeping, HIV target) is indicated by a different color. [file 12977_2019_494_MOESM10_ESM.tif]

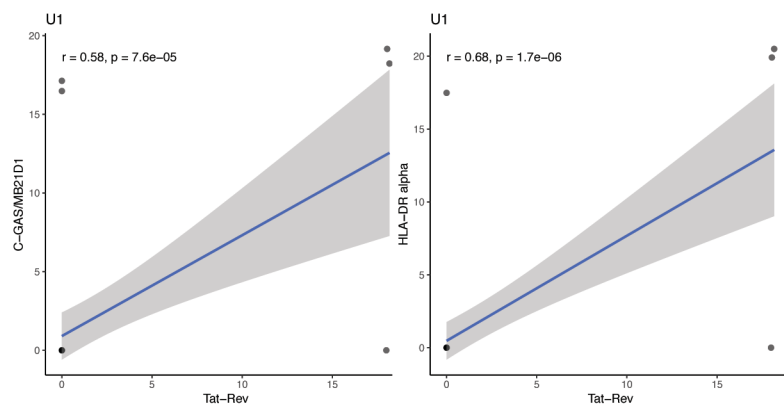

Supplement: Supplementary file 11 — Additional file 11: Fig. S8. False correlations driven by non-detection of cellular and HIV targets. Shown are correlations between Tat-Rev and expression of C-GAS (left panel) and HLA-DR (right panel) in U1 cells. [file 12977_2019_494_MOESM11_ESM.pdf]

**A**

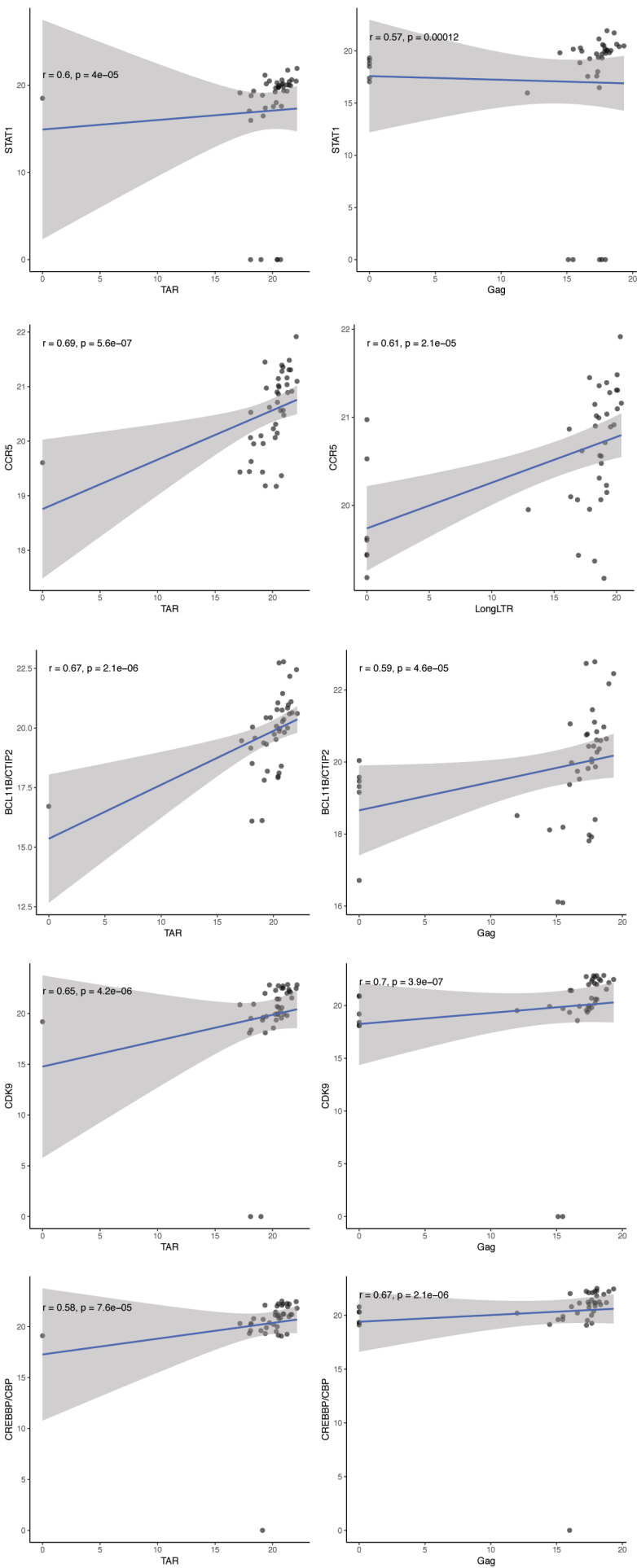

**B**

Activated J-Lat 9.2

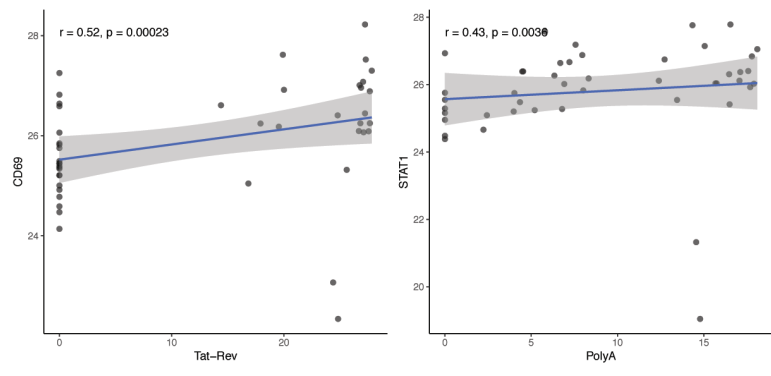

Supplement: Supplementary file 12 — Additional file 12: Fig. S9. Positive correlations between expression of cellular and HIV targets in J-Lat 9.2 (untreated and activated). Positive correlations between cellular and HIV targets in A non-activated J-Lat 9.2 and B activated J-Lat 9.2. R and p values are from Spearman correlations. [file 12977_2019_494_MOESM12_ESM.pdf]

# Untreated vs. activated J-Lat 9.2 cells

● NS ● Log2 FC ● P ● P & Log2 FC

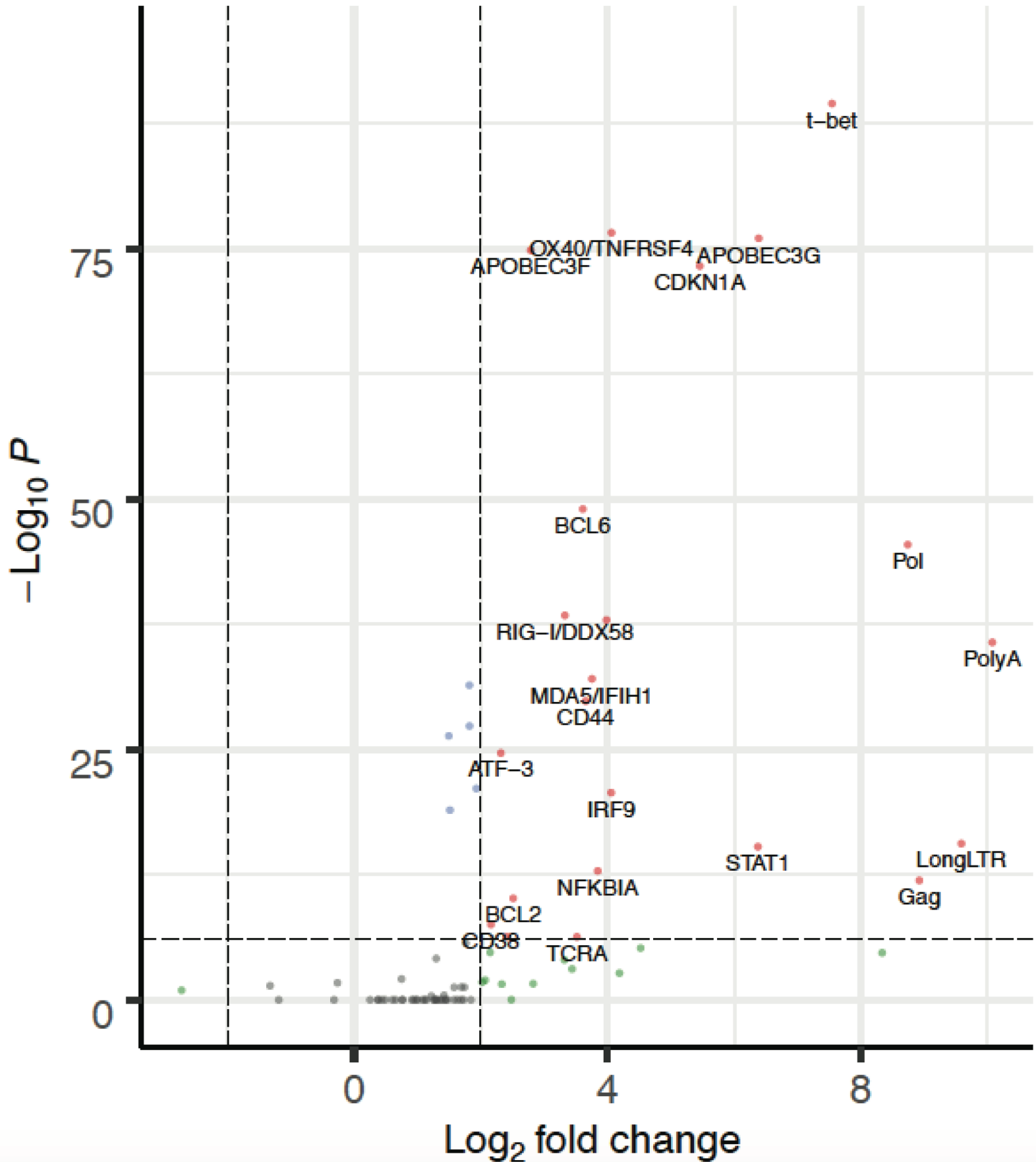

Supplement: Supplementary file 13 — Additional file 13: Fig. S10. Differentially expressed genes in unstimulated vs. activated J-Lat 9.2 cells. Each dot represents a separate gene or HIV target. The X axis represents the log2 fold change and the y-axis denotes the − log10(P value). [file 12977_2019_494_MOESM13_ESM.pdf]
