# Supplementary material for: Heterogeneity in HIV and cellular transcription profiles in cell line models of latent and productive infection: implications for HIV latency
Source: Retrovirology. 2019 Nov 11;16:32. doi: 10.1186/s12977-019-0494-x (PMC6849327; doi:10.1186/s12977-019-0494-x)
Supplement: Supplementary file 3 — Additional file 3: Table S1. caHIV RNA transcript ratios. [file 12977_2019_494_MOESM3_ESM.docx]

| **Table S1. caHIV RNA transcript ratios** | | | | | |  |  |  |  |  |  |  |  |  |  |
| --- | --- | --- | --- | --- | --- | --- | --- | --- | --- | --- | --- | --- | --- | --- | --- |
| cells | RTh/TAR | longLTR/TAR | Pol/TAR | Nef/TAR | polyA/TAR | TatRev/TAR | RTh/longLTR | Pol/longLTR | Nef/longLTR | PolyA/longLTR | TatRev/longLTR | Nef/Pol | PolyA/Pol | PolyA/Nef | TatRev/polyA |
| CD4+ T cells,  median [Min-Max] | 0.012  [0.002-0.019] | 0.097  [0.009-0.290] | 0.025  [0.014-0.035] | 0.006  [0.003-0.008] | 0.012  [0.004-0.070] | 0.0004  [0.000-0.001] | 0.145  [0.031-0.264] | 0.379  [0.309-0.427] | 0.097  [0.054-0.163] | 0.143  [0.043-7.753] | 0.005  [0.000-0.013] | 0.277  [0.147-0.473] | 0.316  [0.226-0.417] | 1.041  [0.611-4.157] | 0.039  [0.000-0.064] |
| PBMC,  median [Min-Max] | 0.028  [0.009-0.195] | 0.077  [0.010-0.351] | · | 0.011  [0.005-0.081] | 0.006  [0.002-0.038] | 0.0002  [0.000-0.003] | 0.356  [0.207-2.524] | · | 0.230  [0.062-0.733] | 0.124  [0.022-0.670] | 0.001  [0.000-0.090] | · | . | 0.474  [0.303-0.860] | 0.007  [0.000-0.269] |
| U1 | 0.004 | 0.107 | 0.041 | 0.084 | 0.057 | 0.016 | 0.038 | 0.386 | 0.789 | 0.532 | 0.150 | 2.043 | 1.378 | 0.674 | 0.282 |
| ACH2 | 0.096 | 0.426 | 0.355 | 0.311 | 0.590 | 0.155 | 0.226 | 0.833 | 0.729 | 1.386 | 0.364 | 0.876 | 1.664 | 1.900 | 0.263 |
| J-lat 6.3 | 0.006 | 0.089 | 0.022 | · | 0.924 | 0.023 | 0.065 | 0.245 | · | 10.417 | 0.255 | · | 42.553 | · | 0.025 |
| J-lat 8.4 | 0.010 | 0.014 | 0.005 | · | 0.767 | 0.001 | 0.717 | 0.333 | · | 56.667 | 0.080 | · | 170.000 | · | 0.001 |
| J-lat 15.4 | 0.033 | 0.029 | 0.038 | · | 0.752 | 0.002 | 1.132 | 1.311 | · | 25.894 | 0.077 | · | 19.747 | · | 0.003 |
| J-lat 9.2 | 0.014 | 0.264 | 0.218 | · | 0.898 | 0.030 | 0.055 | 0.828 | · | 3.409 | 0.113 | · | 4.116 | · | 0.033 |
| J-lat 5A8 | 0.022 | 0.025 | 0.015 | · | 0.214 | 0.002 | 0.862 | 0.606 | · | 8.515 | 0.060 | · | 14.045 | · | 0.007 |
| Activated J-lat 9.2 | 0.005 | 0.582 | 0.402 | · | 0.624 | 0.092 | 0.009 | 0.691 | · | 1.072 | 0.158 | · | 14.045 | · | 0.148 |
| Activated J-lat 5A8 | 0.005 | 0.190 | 0.062 | · | 0.151 | 0.026 | 0.024 | 0.325 | · | 0.797 | 0.138 | · | 2.449 | · | 0.173 |
| 8E5 | 0.002 | 0.166 | 0.162 | 0.106 | 0.105 | 0.017 | 0.015 | 0.977 | 0.638 | 0.635 | 0.103 | 0.653 | 1.552 | 0.995 | 0.162 |
| Activated CD4+ T cells,  median [Min-Max] | 0.009  [0.001-0.047] | 0.240  [0.077-0.587] | · | · | 0.201  [0.025-0.492] | 0.021  [0.003-0.054] | 0.053  [0.012-0.151] | · | · | 0.854  [0.202-1.966] | 0.074  [0.033-0.209] | · | 2.449 | · | 0.106  [0.034-0.255] |
