# Supplementary material for: Heterogeneity in HIV and cellular transcription profiles in cell line models of latent and productive infection: implications for HIV latency
Source: Retrovirology. 2019 Nov 11;16:32. doi: 10.1186/s12977-019-0494-x (PMC6849327; doi:10.1186/s12977-019-0494-x)
Supplement: Supplementary file 4 — Additional file 4: Table S2. Complete assay panel. [file 12977_2019_494_MOESM4_ESM.docx]

**Table S2. Complete assay panel**

| **Gene Name** | **Full gene name** | **Aliases** | **Category** | **Catalog #** | **Gene Name** | **Full gene name** | **Aliases** | **Category** | **Catalog #** |
| --- | --- | --- | --- | --- | --- | --- | --- | --- | --- |
| **APOBEC3F** | apolipoprotein B mRNA editing enzyme catalytic subunit 3F | A3F; ARP8; BK150C2.4.MRNA; KA6 | Antiviral/Restriction Factor | Hs01665324_m1 | **POLR2A** | RNA polymerase II subunit A | hRPB220; hsRPB1; POLR2; POLRA; RPB1; RPBh1; RpIILS; RPO2; RPOL2 | HIV transcription/latency | Hs00172187_m1 |
| **APOBEC3G** | apolipoprotein B mRNA editing enzyme catalytic subunit 3G | A3G; ARCD; ARP-9; ARP9; bK150C2.7; CEM-15; CEM15; dJ494G10.1; MDS019 | Antiviral/Restriction Factor | Hs00222415_m1 | **PRDM1/Blimp-1** | PR domain 1 | BLIMP1; PRDI-BF1 | HIV transcription/latency | Hs00153357_m1 |
| **BST2/Tetherin** | bone marrow stromal cell antigen 2 | CD317; Tetherin | Antiviral/Restriction Factor | Hs01561315_m1 | **PRMT6** | protein arginine methyltransferase 6 | HRMT1L6 | HIV transcription/latency | Hs00250803_s1 |
| **CDKN1A (p21)** | cyclin dependent kinase inhibitor 1A | CAP20; CDKN1; CIP1; MDA-6; P21; p21CIP1; SDI1; WAF1 | Antiviral/Restriction Factor | Hs00355782_m1 | **PSIP1** | PC4 and SFRS1 interacting protein 1 | DFS70; LEDGF; p52; p75; PAIP; PSIP2 | HIV transcription/latency | Hs01045703_m1 |
| **C-GAS/MB21D1** | Mab-21 domain containing 1 | C6orf150; cGAS; h-cGAS | Antiviral/Restriction Factor | Hs00403553_m1 | **PTB/PTBP1** | polypyrimidine tract binding protein 1 | HNRNP-I; HNRNPI; HNRPI; pPTB; PTB; PTB-1; PTB-T; PTB2; PTB3; PTB4 | HIV transcription/latency | Hs00914687_g1 |
| **IFNA1** | interferon alpha 1 | IFL; IFN; IFN-ALPHA; IFN-alphaD; IFNA13; IFNA@ | Antiviral/Restriction Factor | Hs00256882_s1 | **RPS6KA3** | ribosomal protein S6 kinase A3 | CLS; HU-3; ISPK-1; MAPKAPK1B; MRX19; p90-RSK2; pp90RSK2; RSK; RSK2; S6K-alpha3 | HIV transcription/latency | Hs00177936_m1 |
| **IRF9** | interferon regulatory factor 9 | IRF-9; ISGF3; ISGF3G; p48 | Antiviral/Restriction Factor | Hs00196051_m1 | **SF2/ASF (SRSF1)** | serine and arginine rich splicing factor 1 | ASF; SF2; SF2p33; SFRS1; SRp30a | HIV transcription/latency | Hs00199471_m1 |
| **MDA5/IFIH1** | interferon induced with helicase C domain 1 | AGS7; Hlcd; IDDM19; MDA-5; MDA5; RLR-2; SGMRT1 | Antiviral/Restriction Factor | Hs01070332_m1 | **SLC16A6** | solute carrier family 16 member 6 | MCT6; MCT7 | HIV transcription/latency | Hs00190779_m1 |
| **PAF-1** | PAF1 homolog, Paf1/RNA polymerase II complex component | F23149_1; PD2 | Antiviral/Restriction Factor | Hs00219496_m1 | **Sp1** | Sp1 transcription factor |  | HIV transcription/latency | Hs00916521_m1 |
| **RIG-I/DDX58** | DEXD/H-box helicase 58 | RIG-I; RIGI; RLR-1; SGMRT2 | Antiviral/Restriction Factor | Hs01061444_m1 | **SUV39H1** | suppressor of variegation 3-9 homolog 1 | H3-K9-HMTase 1; KMT1A; MG44; SUV39H | HIV transcription/latency | Hs00957892_m1 |
| **SAMHD1** | SAM and HD domain containing deoxynucleoside triphosphate triphosphohydrolase 1 | CHBL2; DCIP; HDDC1; MOP-5; SBBI88 | Antiviral/Restriction Factor | Hs00210019_m1 | **TNFRSF8** | TNF receptor superfamily member 8 | CD30; D1S166E; Ki-1 | HIV transcription/latency | Hs00174277_m1 |
| **SLFN11** | schlafen family member 11 | SLFN8/9 | Antiviral/Restriction Factor | Hs00536981_m1 | **TRBP** | TARBP2, RISC loading complex RNA binding subunit | LOQS; TRBP; TRBP1; TRBP2 | HIV transcription/latency | Hs00998379_m1 |
| **STAT1** | signal transducer and activator of transcription 1 | CANDF7; IMD31A; IMD31B; IMD31C; ISGF-3; STAT91 | Antiviral/Restriction Factor | Hs01013996_m1 | **YY1** | YY1 transcription factor | DELTA; INO80S; NF-E1; UCRBP; YIN-YANG-1 | HIV transcription/latency | Hs00231533_m1 |
| **STING/TMEM173** | transmembrane protein 173 | ERIS; hMITA; hSTING; MITA; MPYS; NET23; SAVI; STING | Antiviral/Restriction Factor | Hs00736958_m1 | **GAPDH** | glyceraldehyde-3-phosphate dehydrogenase | G3PD; GAPD; HEL-S-162eP | Housekeeping | Hs99999905_m1 |
| **TRIM5** | tripartite motif containing 5 | RNF88; TRIM5alpha | Antiviral/Restriction Factor | Hs01552559_m1 | **PPIA** | peptidylprolyl isomerase A | CYPA; CYPH; HEL-S-69p | Housekeeping | Hs99999904_m1 |
| **Gag** |  |  | HIV target | Custom | **RPL13a** | ribosomal protein L13a | L13A; TSTA1 | Housekeeping | Hs04194366_g1 |
| **Long LTR** |  |  | HIV target | Custom | **BCL6** | B-cell CLL/lymphoma 6 | BCL5; BCL6A; LAZ3; ZBTB27; ZNF51 | T cell phenotype/function | Hs00153368_m1 |
| **Nef** |  |  | HIV target | Custom | **beta7 integrin/ITGB7** | integrin subunit beta 7 |  | T cell phenotype/function | Hs01565750_m1 |
| **Pol** |  |  | HIV target | Custom | **CASP3** | caspase 3 | CPP32; CPP32B; SCA-1 | T cell phenotype/function | Hs00234387_m1 |
| **PolyA** |  |  | HIV target | Custom | **CCR5** | C-C motif chemokine receptor 5 (gene/pseudogene) | CC-CKR-5; CCCKR5; CCR-5; CD195; CKR-5; CKR5; CMKBR5; IDDM22 | T cell phenotype/function | Hs99999149_s1 |
| **TAR** |  |  | HIV target | Custom | **CCR7** | C-C motif chemokine receptor 7 | BLR2; CC-CKR-7; CCR-7; CD197; CDw197; CMKBR7; EBI1 | T cell phenotype/function | Hs01013469_m1 |
| **Tat-Rev** |  |  | HIV target | Custom | **CD103** | integrin subunit alpha E | ITGAE;CD103; HUMINAE | T cell phenotype/function | Hs01025372_m1 |
| **CD28** | CD28 molecule | Tp44 | HIV transcription/latency | Hs01007422_m1 | **CD25/IL2RA** | interleukin 2 receptor subunit alpha | CD25; IDDM10; IL2R; IMD41; p55; TCGFR | T cell phenotype/function | Hs00907779_m1 |
| **ATF-3** | activating transcription factor 3 |  | HIV transcription/latency | Hs00231069_m1 | **CD3 delta** | CD3d molecule | CD3-DELTA; IMD19; T3D | T cell phenotype/function | Hs00174158_m1 |
| **BCL11B/CTIP2** | B-cell CLL/lymphoma 11B | ATL1; ATL1-alpha; ATL1-beta; ATL1-delta; ATL1-gamma; CTIP-2; CTIP2; hRIT1-alpha; RIT1; ZNF856B | HIV transcription/latency | Hs01102259_m1 | **CD38** | CD38 molecule | ADPRC 1; ADPRC1 | T cell phenotype/function | Hs01120071_m1 |
| **BCL2** | BCL2, apoptosis regulator | Bcl-2; PPP1R50 | HIV transcription/latency | Hs00608023_m1 | **CD4** | CD4 molecule | CD4mut | T cell phenotype/function | Hs01065472_m1 |
| **CCNL2/cyclin L2** | cyclin L2 | ANIA-6B; CCNM; CCNS; HCLA-ISO; HLA-ISO; PCEE; SB138 | HIV transcription/latency | Hs01085988_m1 | **CD44** | CD44 molecule (Indian blood group) | CDW44; CSPG8; ECMR-III; HCELL; HUTCH-I; IN; LHR; MC56; MDU2; MDU3; MIC4; Pgp1 | T cell phenotype/function | Hs01075861_m1 |
| **CDK13** | cyclin dependent kinase 13 | CDC2L; CDC2L5; CHED; hCDK13 | HIV transcription/latency | Hs00243534_m1 | **CD69** | CD69 molecule | AIM; BL-AC/P26; CLEC2C; EA1; GP32/28; MLR-3 | T cell phenotype/function | Hs00934033_m1 |
| **CDK7** | cyclin dependent kinase 7 | CAK; CAK1; CDKN7; HCAK; MO15; p39MO15; STK1 | HIV transcription/latency | Hs00361486_m1 | **CD8a** | CD8a molecule | CD8; Leu2; MAL; p32 | T cell phenotype/function | Hs00233520_m1 |
| **CDK9** | cyclin dependent kinase 9 | C-2k; CDC2L4; CTK1; PITALRE; TAK | HIV transcription/latency | Hs00176222_m1 | **CTLA4** | cytotoxic T-lymphocyte associated protein 4 | ALPS5; CD; CD152; CELIAC3; CTLA-4; GRD4; GSE; IDDM12 | T cell phenotype/function | Hs00175480_m1 |
| **CEBPB** | CCAAT/enhancer binding protein beta | C/EBP-beta; IL6DBP; NF-IL6; TCF5 | HIV transcription/latency | Hs00270923_s1 | **CXCR4** | C-X-C motif chemokine receptor 4 | CD184; D2S201E; FB22; HM89; HSY3RR; LAP-3; LAP3; LCR1; LESTR; NPY3R; NPYR; NPYRL; NPYY3R; WHIM; WHIMS | T cell phenotype/function | Hs00237052_m1 |
| **CREBBP/CBP** | CREB binding protein | CBP; KAT3A; RSTS | HIV transcription/latency | Hs00231733_m1 | **FAS** | Fas cell surface death receptor | ALPS1A; APO-1; APT1; CD95; FAS1; FASTM; TNFRSF6 | T cell phenotype/function | Hs00907754_m1 |
| **EGR1** | early growth response 1 | AT225; G0S30; KROX-24; NGFI-A; TIS8; ZIF-268; ZNF225 | HIV transcription/latency | Hs00152928_m1 | **FosB** | FosB proto-oncogene, AP-1 transcription factor subunit | AP-1; G0S3; GOS3; GOSB | T cell phenotype/function | Hs00171851_m1 |
| **ELL** | elongation factor for RNA polymerase II | C19orf17; ELL1; MEN; PPP1R68 | HIV transcription/latency | Hs00197950_m1 | **GATA3** | GATA binding protein 3 | HDR; HDRS | T cell phenotype/function | Hs00231122_m1 |
| **EP300** | E1A binding protein p300 | KAT3B; p300; RSTS2 | HIV transcription/latency | Hs00914223_m1 | **HLA-DR alpha** | major histocompatibility complex, class II, DR alpha | HLA-DRA1; MLRW | T cell phenotype/function | Hs00219575_m1 |
| **EZH2** | enhancer of zeste 2 polycomb repressive complex 2 subunit | ENX-1; ENX1; EZH1; EZH2b; KMT6; KMT6A; WVS; WVS2 | HIV transcription/latency | Hs00544830_m1 | **IFI16** | interferon gamma inducible protein 16 | IFNGIP1; PYHIN2 | T cell phenotype/function | Hs00194261_m1 |
| **G9a/EHMT2** | euchromatic histone lysine methyltransferase 2 | BAT8; C6orf30; G9A; GAT8; KMT1C; NG36 | HIV transcription/latency | Hs00198710_m1 | **Ki67/MKI67** | marker of proliferation Ki-67 | KIA; MIB-; MIB-1; PPP1R105 | T cell phenotype/function | Hs01032443_m1 |
| **HDAC5** | histone deacetylase 5 | HD5; NY-CO-9 | HIV transcription/latency | Hs00608366_m1 | **LCK** | LCK proto-oncogene, Src family tyrosine kinase | IMD22; LSK; p56lck; pp58lck; YT16 | T cell phenotype/function | Hs00178427_m1 |
| **HTATSF1** | HIV-1 Tat specific factor 1 | dJ196E23.2; TAT-SF1; TATSF1 | HIV transcription/latency | Hs00205619_m1 | **p53** | tumor protein p53 | BCC7; LFS1; P53; TRP53 | T cell phenotype/function | Hs01034249_m1 |
| **MATR3** | matrin 3 | ALS21; MPD2; VCPDM | HIV transcription/latency | Hs00251579_m1 | **PD-1 (PDCD1)** | programmed cell death 1 | CD279; hPD-1; hPD-l; hSLE1; PD-1; PD1; SLEB2 | T cell phenotype/function | Hs01550088_m1 |
| **NELFA/WHS2** | negative elongation factor complex member A | NELF-A; P/OKcl.15; WHSC2 | HIV transcription/latency | Hs00171805_m1 | **PTPRC/CD45exon3-4** | protein tyrosine phosphatase, receptor type C | B220; CD45; CD45R; GP180; L-CA; LCA; LY5; T200 | T cell phenotype/function | Hs00174541_m1 |
| **NFATC1** | nuclear factor of activated T-cells 1 | NF-ATC; NF-ATc1.2; NFAT2; NFATc | HIV transcription/latency | Hs00542678_m1 | **RORC** | RAR related orphan receptor C | IMD42; NR1F3; RORG; RZR-GAMMA; RZRG; TOR | T cell phenotype/function | Hs01076122_m1 |
| **NFKB1** | nuclear factor kappa B subunit 1 | CVID12; EBP-1; KBF1; NF-kappa-B; NF-kappaB; NF-kB1; NFkappaB; NFKB-p105; NFKB-p50; p105; p50 | HIV transcription/latency | Hs00765730_m1 | **SF3B2** | splicing factor 3b subunit 2 | Cus1; SAP145; SF3b1; SF3B145; SF3b150 | T cell phenotype/function | Hs00199190_m1 |
| **NFKBIA** | NFKB inhibitor alpha | IKBA; MAD-3; NFKBI | HIV transcription/latency | Hs00153283_m1 | **t-bet** | T-box 21 | T-bet; T-PET; TBET; TBLYM | T cell phenotype/function | Hs00203436_m1 |
| **OX40/TNFRSF4** | TNF receptor superfamily member 4 | ACT35; CD134; IMD16; OX40; TXGP1L | HIV transcription/latency | Hs00533968_m1 | **TCF7** | transcription factor 7 (T-cell specific, HMG-box) | TCF-1 | T cell phenotype/function | Hs01556515_m1 |
| **PAPOLA** | poly(A) polymerase alpha | PAP | HIV transcription/latency | Hs04276840_m1 | **TCRA** | T cell receptor alpha variable 20 | hDV102S1; IMD7; TCRA; TCRAV20S1; TCRAV30S1; TRA; TRCA | T cell phenotype/function | Hs00948942_m1 |
| **PBAF/SMARCA4** | SWI/SNF related, matrix associated, actin dependent regulator of chromatin, subfamily a, member 4 | BAF190; BAF190A; BRG1; CSS4; hSNF2b; MRD16; RTPS2; SNF2; SNF2L4; SNF2LB; SWI2 | HIV transcription/latency | Hs00231324_m1 | **TGFB1** | transforming growth factor beta 1 | CED; DPD1; LAP; TGFB; TGFbeta | T cell phenotype/function | Hs00998133_m1 |
| **PCAF** | lysine acetyltransferase 2B | CAF; P/CAF; PCAF | HIV transcription/latency | Hs00187332_m1 | **TIGIT** | T-cell immunoreceptor with Ig and ITIM domains | VSIG9; VSTM3; WUCAM | T cell phenotype/function | Hs00545087_m1 |
