# Supplementary material for: Heterogeneity in HIV and cellular transcription profiles in cell line models of latent and productive infection: implications for HIV latency
Source: Retrovirology. 2019 Nov 11;16:32. doi: 10.1186/s12977-019-0494-x (PMC6849327; doi:10.1186/s12977-019-0494-x)
Supplement: Supplementary file 7 — Additional file 7: Table S3. Number of single-cells analyzed across cell lines. [file 12977_2019_494_MOESM7_ESM.docx]

**Table S3.** **Number of single-cells analyzed across cell lines**

|  | **Batch 1** | **Batch 2** | **Batch 3** |
| --- | --- | --- | --- |
| **8E5** | 35 | 0 | 9 |
| **J-Lat (Stim)** | 0 | 35 | 10 |
| **J-Lat** | 0 | 30 | 11 |
| **ACH2** | 0 | 0 | 40 |
| **U1** | 33 | 0 | 7 |
